# Properties of genes essential for mouse development

**DOI:** 10.1371/journal.pone.0178273

**Published:** 2017-05-31

**Authors:** Mitra Kabir, Ana Barradas, George T. Tzotzos, Kathryn E. Hentges, Andrew J. Doig

**Affiliations:** 1 Faculty of Biology, Medicine, and Health, University of Manchester, Manchester, United Kingdom; 2 Manchester Institute of Biotechnology and Department of Chemistry, Faculty of Science and Engineering, The University of Manchester, Manchester, United Kingdom; 3 Department of Agriculture, Food and Environmental Sciences, Marche Polytechnic University, Ancona, Italy; Tianjin University, CHINA

## Abstract

Essential genes are those that are critical for life. In the specific case of the mouse, they are the set of genes whose deletion means that a mouse is unable to survive after birth. As such, they are the key minimal set of genes needed for all the steps of development to produce an organism capable of life *ex utero*. We explored a wide range of sequence and functional features to characterise essential (lethal) and non-essential (viable) genes in mice. Experimental data curated manually identified 1301 essential genes and 3451 viable genes. Very many sequence features show highly significant differences between essential and viable mouse genes. Essential genes generally encode complex proteins, with multiple domains and many introns. These genes tend to be: long, highly expressed, old and evolutionarily conserved. These genes tend to encode ligases, transferases, phosphorylated proteins, intracellular proteins, nuclear proteins, and hubs in protein-protein interaction networks. They are involved with regulating protein-protein interactions, gene expression and metabolic processes, cell morphogenesis, cell division, cell proliferation, DNA replication, cell differentiation, DNA repair and transcription, cell differentiation and embryonic development. Viable genes tend to encode: membrane proteins or secreted proteins, and are associated with functions such as cellular communication, apoptosis, behaviour and immune response, as well as housekeeping and tissue specific functions. Viable genes are linked to transport, ion channels, signal transduction, calcium binding and lipid binding, consistent with their location in membranes and involvement with cell-cell communication. From the analysis of the composite features of essential and viable genes, we conclude that essential genes tend to be required for intracellular functions, and viable genes tend to be involved with extracellular functions and cell-cell communication. Knowledge of the features that are over-represented in essential genes allows for a deeper understanding of the functions and processes implemented during mammalian development.

## Introduction

Essential genes are those whose presence is imperative for the survival of an organism. However, the complete set of genes that are absolutely vital to sustain life are still unknown for most organisms [1]. In mammals, knowledge of essential genes is required to understand development, maintenance of major cellular processes and tissue-specific functions that are crucial for life. As such, essential genes are the key minimal set of genes needed for all steps of development. Genes that are not needed for development are termed non-essential or viable genes. Mammalian essential genes can be identified using experimental techniques [2], which include single gene knockouts [3–5], conditional knockouts [6, 7], forward genetic screens [8], RNA interference [9, 10], and transposon mutagenesis [11]. Though these experimental methods are the gold standard, they are time consuming and expensive. Nevertheless, major programs are currently underway to systematically knockout every mouse gene and characterise the resulting phenotypes [12]. An initial set of essential genes has been identified through these experimental approaches [13, 14].

These recent data offer a valuable resource to help understand which processes are critical for mammalian development and to discover what makes a gene essential or viable. We hypothesised that essential and viable genes are distinguishable by various attributes. We explored a wide range of sequence and functional features of mouse genes in order to characterise essential and viable genes in mammals. We have discovered numerous gene and protein features that vary significantly between essential and viable genes in mouse, some of which were previously found to be associated with essentiality in *E*. *coli* [15, 16], *S*. *cerevisiae* [17–19], mouse [20] and human [21]. These features thus reveal the key genetic functions required for development in mammals.

## Results

### Datasets

The Mouse Genome Informatics (MGI) database [22] incorporates published gene data on mouse knockout phenotypes. We collected a total of 1,271 essential and 4,378 viable mouse genes from MGI (accessed on 1 November, 2013), based on phenotype annotations of null alleles of targeted deletion knockout mice. Mutant phenotypes generated from other experimental methods were not included in our dataset, since we could not exclude the possibility that essential genes might have hypomorphic alleles with viable phenotypes in gene trap, knockdown, or chemical mutagenesis experiments. We considered a gene as essential if it produced lethality in either the heterozygous or homozygous state, and did not differentiate between these two types of genes in the dataset. We defined essential genes as those that are required for an animal to survive past post-natal day 3. A total of 1,335 genes had both ‘essential’ and ‘viable’ annotations in the MGI database so were individually checked in the literature. We further manually checked each gene to ensure that our datasets contained only protein-coding genes, to allow for an analysis of features specific to protein function. This resulted in a total dataset of 1,301 essential and 3,451 viable mouse genes (S1 Data and S2 Data).

The proteins encoded by these essential and viable genes share significant levels of sequence identity. A protein sequence dataset is considered redundant if it includes a pair of proteins that are highly similar or homologous. The presence of redundancy is a barrier in using a dataset effectively as it increases the size of the dataset; also it could potentially create bias towards any conclusions drawn from the overall analysis using the dataset due to the over-representation of similar features. This problem can be overcome by removing redundant proteins from the dataset until all the proteins in the dataset share sequence similarity less than a predefined threshold. We therefore used Leaf [23] to remove redundant proteins from our datasets. We generated four culled or non-redundant essential and viable datasets from our original dataset, where the sequence similarity between all proteins is less than a threshold of 20%, 40%, 60% and 80%, respectively (Table 1).

**Table 1:** Numbers of essential and viable proteins/genes in the non-redundant datasets.

Sequence Identity Cut-Off	Number of Essential Proteins	Number of Viable Proteins
Non-culled (Complete Set)	1301	3451
20%	479	1017
40%	961	2302
60%	1215	3106
80%	1291	3391

### Analysis of genomic features

The functionality of a gene may rely on its inherent sequence features at the genomic level. Analysing these gene sequence based features may provide valuable insights into their contributions to gene essentiality.

#### GC content, gene length, and transcript diversity

**Fig 1:**
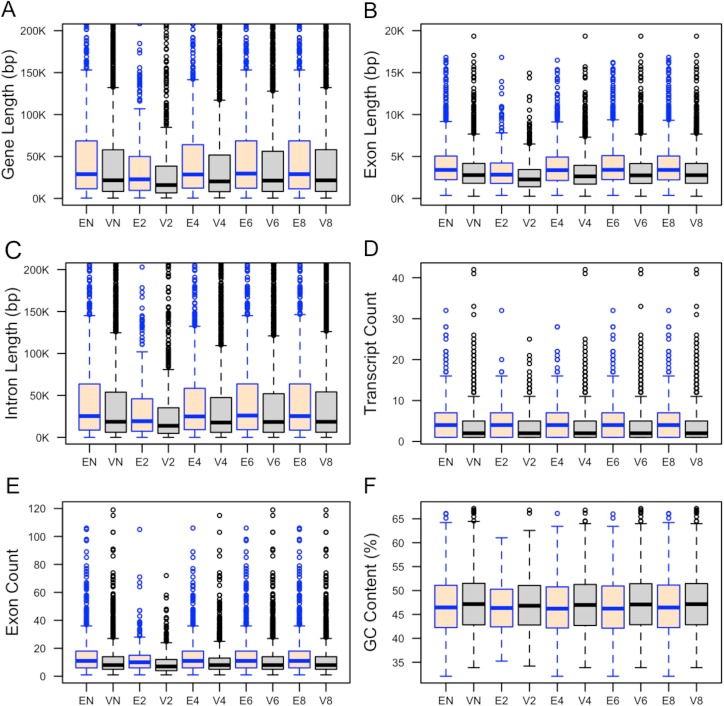
**Distributions of the (A) total gene length, (B) total length of exons, (C) total length of introns, (D) number of transcripts, (E) number of exons, and (F) percentage of GC content in essential and viable genes.** Here, EN and VN refer to essential and viable genes in the non–culled dataset. Ex and Vx define essential and viable genes in the culled dataset where all coded proteins share sequence similarity less than x0%. In this box plot, the top and bottom of the box denote the upper and lower quartiles; the line inside the box denotes the median; and individual points denote the outliers. Top 5% essential and viable genes with longest gene length (A) and longest introns (F) were excluded from the datasets to make plots more readable.

We anticipated that genomic features such as gene length and GC content could be indicative of gene essentiality, and determined if these features differ between our essential and non-essential gene sets. We found that essential genes tend to be longer in length compared to viable genes (Table 2; Fig 1A). Total gene length is comprised of individual exon and intron lengths. We therefore also measured these features in our datasets, finding that essential genes tend to have longer exons and introns than viable genes (Table 2; Fig 1B and Fig 1C).

**Table 2:** Median gene length, GC contents, number of transcripts, number of exons, exon length and intron length for essential and viable genes. The median value of each feature is reported. p–values are determined from a Mann–Whitney U test. Statistically significant results were evaluated based on the Bonferroni corrected p–value of 0.0083.

Datasets			Gene Sequence Features				
		Gene Length (bp)	GC content (%)	No. of transcripts	No. of exons	Exon length (bp)	Intron length (bp)
**Non-culled**	Essential	28913	46.46	4	11	3398	25341
	Viable	21629	47.16	2	8	2780	18563
	**p-value**	7.9×10^−8^	0.009	4.7×10^−16^	9.4×10^−16^	1.2×10^−22^	2.0×10^−6^
**Culled (20%)**	Essential	22757.5	46.34	4	10	2831	19226
	Viable	15931	46.82	2	7	2263	13761
	**p-value**	5.0×10^−6^	0.091	1.3×10^−10^	6.3×10^−9^	2.2×10^−10^	5.8×10^−5^
**Culled (40%)**	Essential	28548	46.21	4	11	3368	24928
	Viable	20280	46.98	2	8	2632	17667
	**p-value**	1.4×10^−8^	0.009	1.8×10^−15^	1.3×10^−20^	9.1×10^−23^	3.3×10^−7^
**Culled (60%)**	Essential	29601	46.22	4	11	3409	25964
	Viable	21267	47.08	2	8	2746	18310
	**p-value**	1.4×10^−9^	0.001	4.5×10^−17^	2.2×10^−18^	2.7×10^−24^	4.1×10^−8^
**Culled (80%)**	Essential	28936	46.45	4	11	3398	25333
	Viable	21574	47.15	2	8	2767	18534
	**p-value**	1.8×10^−7^	0.013	5.5×10^−16^	1.2×10^−15^	1.2×10^−22^	3.0×10^−6^

We also examined transcript diversity, finding that essential genes tend to have more transcripts than viable genes (Table 2; Fig 1D). To quantify whether or not the number of exons could differentiate between essential and viable genes, the ranking of the number of exons from the longest transcript of each gene was analysed. We found that essential genes are likely to have more exons than viable genes (Table 2; Fig 1E).

When the distributions of GC content in essential and viable genes were examined, we observed that viable genes have a higher percentage of GC content only for the culled dataset where all coded proteins have a sequence identity < 60%; this observation was not statistically significant for other culled datasets (Table 2; Fig 1F).

#### Gene expression

**Fig 2:**
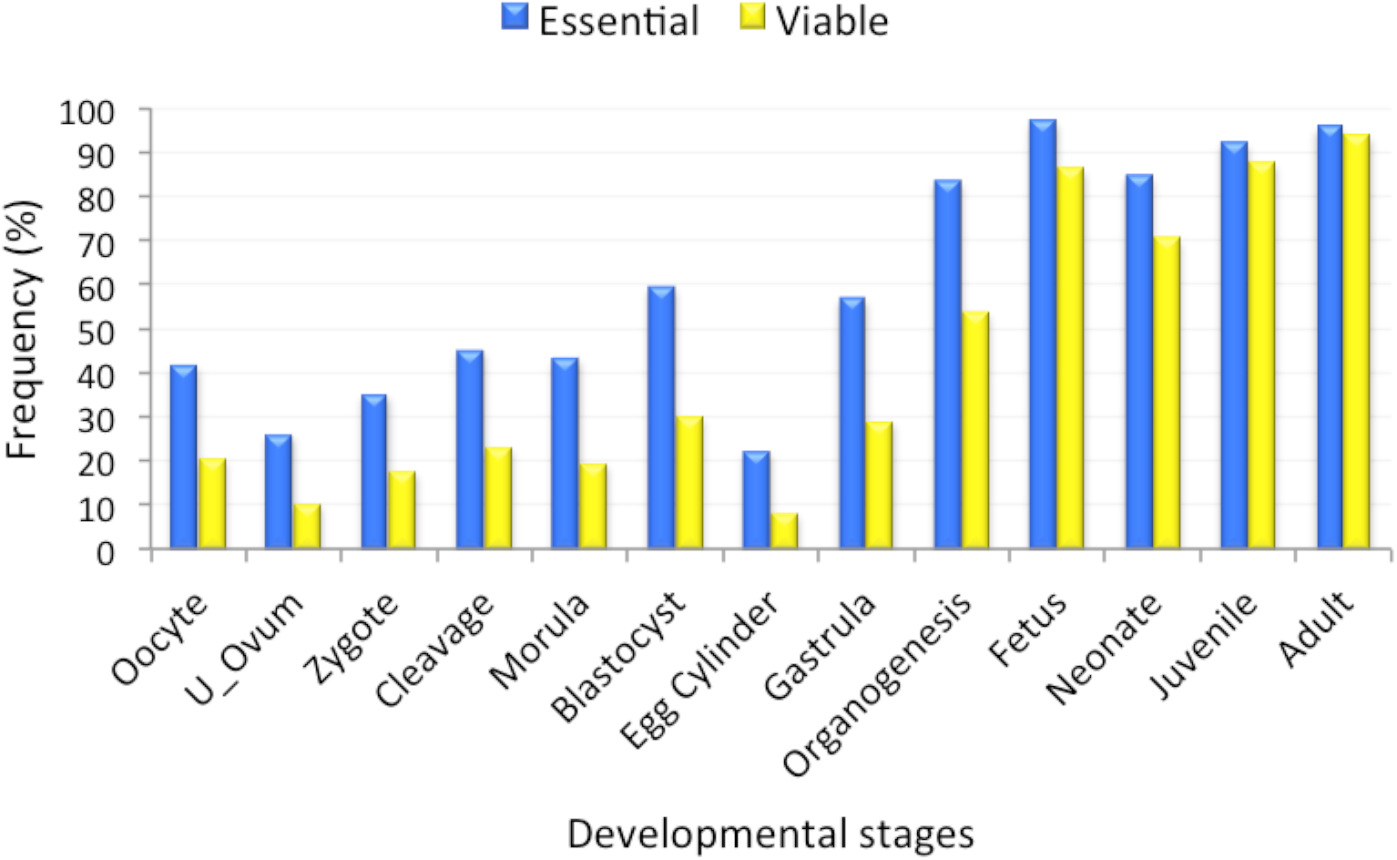
Frequencies (%) of essential and viable mouse genes in the non-culled datasets that are expressed at 13 embryonic developmental stages.

**Fig 3:**
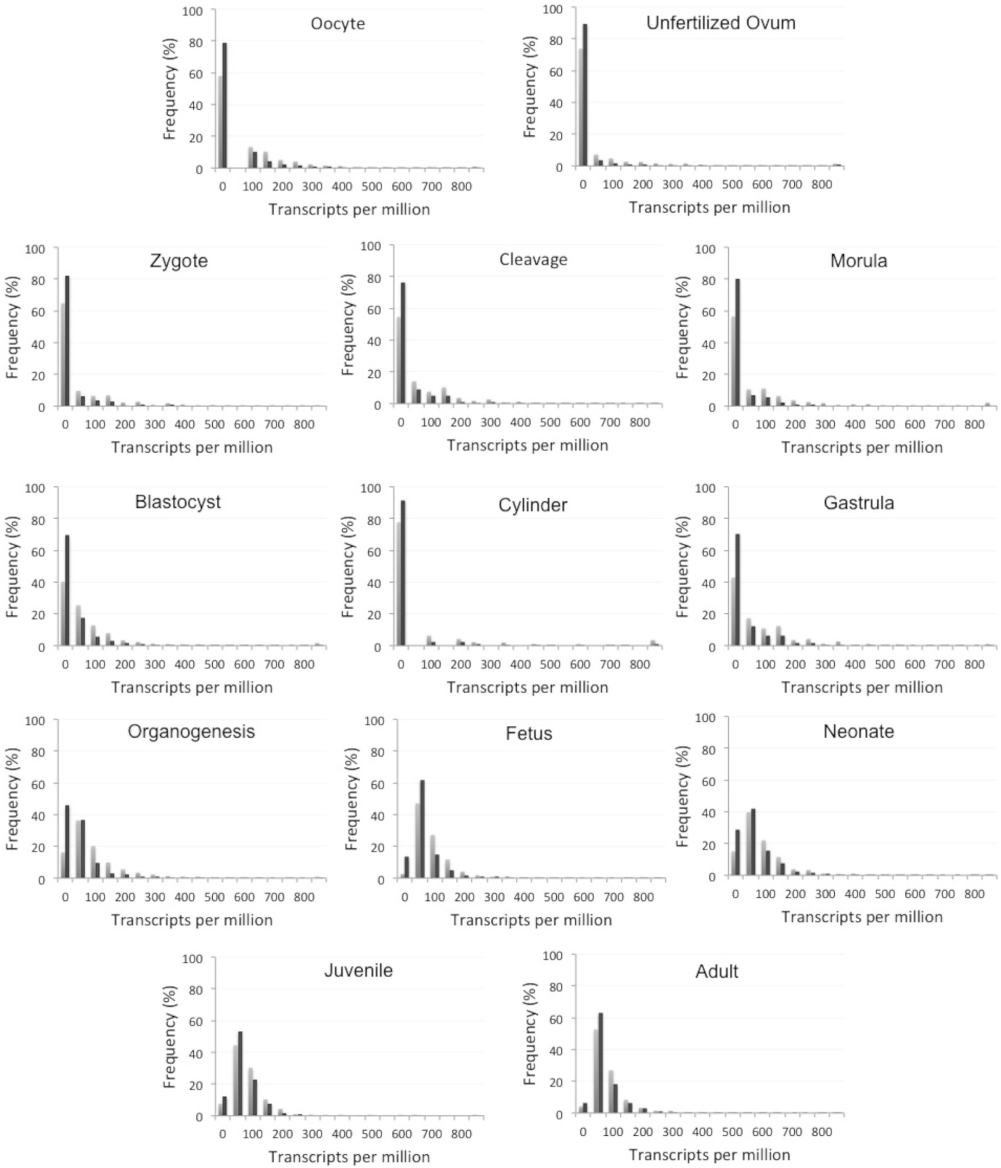
Gene expression distributions of essential and viable genes in the non-culled dataset across 13 stages of mouse development. Here, the bin size is 50.

Examining the temporal specificity of gene expression can identify genes that are active in a particular biological process. We therefore expected that expression could serve as an important indicator of essentiality, as developmentally essential genes should be expressed during embryonic development. We obtained mouse gene expression data for 1,301 essential and 3,409 viable genes from the UniGene database [24] covering 13 developmental stages. Essential genes are more highly expressed than viable genes at every stage of mouse development (Fig 2). However, the *χ*^2^ tests with the Bonferroni correction analysis showed that these differences are not statistically significant at later stages of development (juvenile and adult), as nearly all genes are expressed at those stages (Table 3). Essential genes were found to be highly expressed, whereas viable genes are more likely to be found in the group of genes with zero transcripts present in developmental samples (Fig 3).

**Table 3:** Frequencies of essential versus viable mouse genes expressed at different developmental stages in the non-culled dataset. The p-value for the Bonferroni correction is 0.00385.

Developmental stage	Essential (%)	Viable (%)	p-value
**Oocyte**	42.0	20.9	3.6×10^−36^
**Unfertilized Ovum**	26.3	10.5	5.9×10^−36^
**Zygote**	35.3	17.8	2.9×10^−29^
**Cleavage**	45.5	23.2	3.0×10^−36^
**Morula**	43.6	19.7	2.2×10^−46^
**Blastocyst**	59.8	30.4	1.2×10^−47^
**Egg Cylinder**	22.4	8.3	6.0×10^−35^
**Gastrula**	57.1	29.3	8.1×10^−45^
**Organogenesis**	83.8	54.1	1.0×10^−30^
**Fetus**	97.3	86.4	4.3×10^−4^
**Neonate**	85.0	70.7	5.1×10^−7^
**Juvenile**	92.4	87.8	0.14
**Adult**	95.9	94.1	0.56

#### Evolutionary age

The evolutionary age of a gene represents the time that has passed since the gene evolved from its ancestor, either by duplication or speciation. Studies in bacteria and yeast found essential genes to be evolutionarily more conserved than viable genes [5, 15, 25]. We therefore also expected that gene evolutionary age could be informative for distinguishing mammalian gene essentiality.

**Fig 4:**
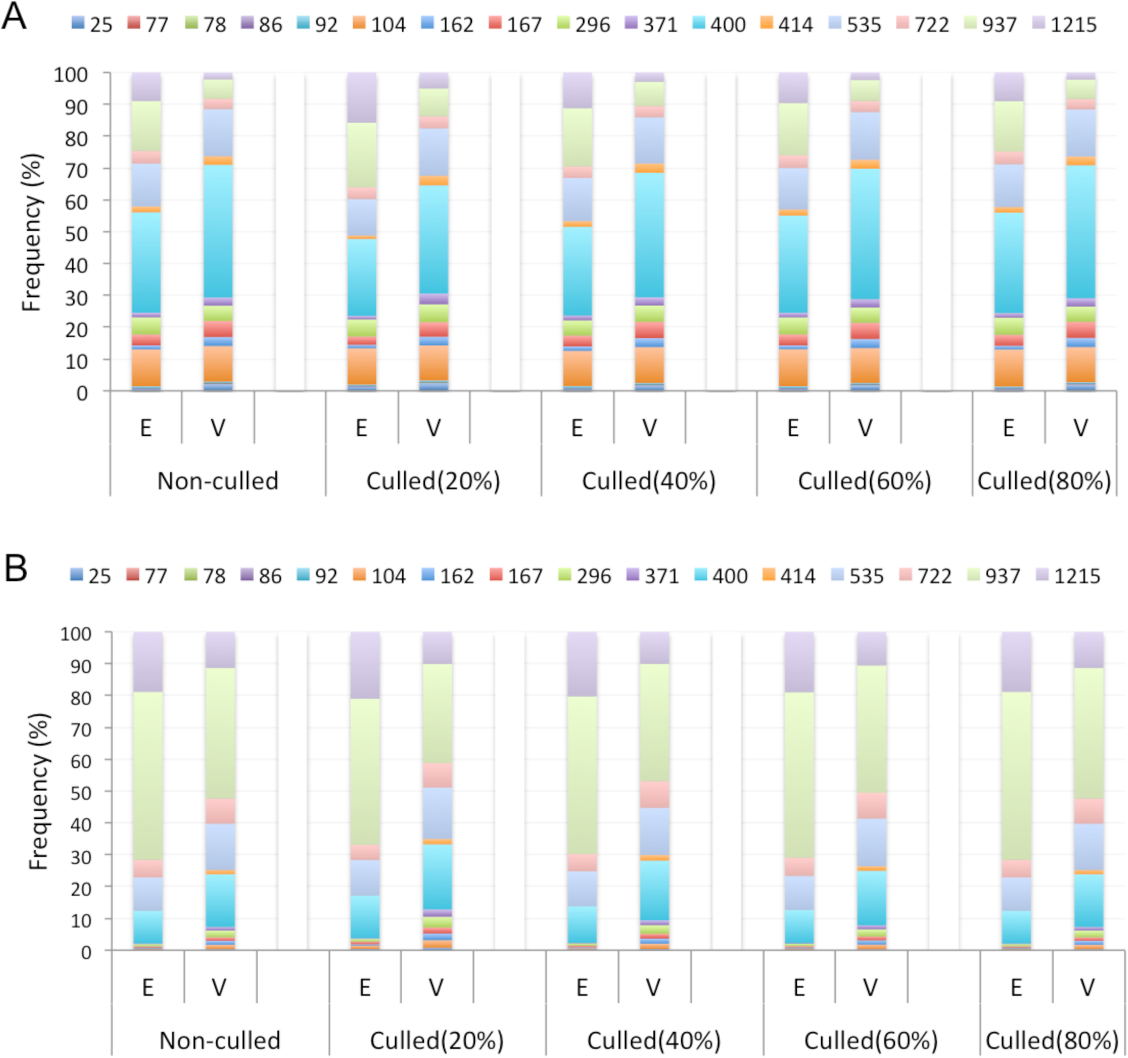
Proportions of essential and viable genes for different age groups. Here, E and V refer to essential and viable genes. Ages of mouse duplicates were calculated based on the MRD event (A) or the DCA (B).

For mammalian genes that have been duplicated, the evolutionary age reported in millions of years ago (MYA) of the duplicate common ancestor (DCA) and the most recent duplication (MRD) event were collected from the Ensembl (release 75) gene trees. For mammalian genes without duplicates, the gene age was determined to be that of the singleton common ancestor (SCA). We observed 16 representative phylogenetic age groups for our mouse genes (Table 4A). We found ages for 1,276 (98.1%) essential and 3,358 (97.3%) viable genes. The oldest genes arose approximately 1215 MYA, whereas the youngest genes belong to the class Murinae arising approximately 25 MYA. We compared the enrichment of essential and viable genes in different age groups. We found that essential genes tend to be older than viable genes for both non-culled and culled datasets (Fig 4). We observed that a significantly greater percentage of essential genes have evolutionary origins of 1215 and 937 MYA, compared to viable genes in the non-culled dataset (Table 4B and Table 4C). The majority of the viable genes arose 400 MYA. Using MRD ages, we found that viable genes are more likely to have ages of 25 and 162 MYA (Table 4B). We further observed a significantly greater percentage of viable genes that have DCA ages arising at 296, 371, 414 and 535 MYA (Table 4C). We found similar trends for the culled datasets, which further confirms that genes essential for mouse development are more evolutionarily ancient.

**Table 4:** Gene ages. **(**A) Phylogenetic age groups in million years ago (MYA) retrieved from the Ensembl (release 75) gene trees. (B) Essential versus viable mouse genes frequencies for different MRD+SCA age groups. (C) Essential versus viable mouse genes frequencies for different DCA+SCA age groups. These results are observed for the non-culled dataset. Here, the Bonferroni corrected p-value in the Chi-squared test is 0.003125.

A		B			C		
Taxon or Age Group	Age (MYA)	Essential (%)	Viable (%)	p-value	Essential (%)	Viable (%)	p-value
Murinae	25	0.63	2.17	3.7×10^−4^	0.16	0.36	0.27
Rodentia	77	0.00	0.09	0.286	0.00	0.00	0
Sciurognathi	78	0.00	0.06	0.383	0.00	0.00	0
Glires	86	0.24	0.06	0.104	0.00	0.00	0
Euarchontoglires	92	0.55	0.51	0.858	0.00	0.00	0
Eutheria	104	11.60	11.23	0.737	0.39	1.36	4.5×10^−3^
Theria	162	1.25	2.80	2.3×10^−3^	0.31	1.24	4.2×10^−3^
Mammalia	167	3.45	5.03	0.025	0.39	1.13	0.019
Amniota	296	5.41	4.79	0.403	0.70	2.10	1.1×10^−3^
Tetrapoda	371	1.41	2.56	0.020	0.16	1.13	1.4×10^−3^
Euteleostomi	400	31.58	41.69	8.3×10^−7^	10.34	16.58	7.8×10^−7^
Sarcopterygii	414	1.80	2.74	0.070	0.00	1.36	2.9×10^−5^
Vertebrata	535	13.48	14.80	0.290	10.42	14.51	6.5×10^−4^
Chordata	722	4.00	3.25	0.219	5.60	7.94	8.3×10^−3^
Bilateria	937	15.67	6.08	4.8×10^−23^	52.72	41.01	7.8×10^−8^
Opisthokonta	1215	8.93	2.14	6.6×10^−25^	18.82	11.28	3.1×10^−10^

### Analysis of protein features

Prior research established that different physical, functional and evolutionary properties of proteins can facilitate the prediction of gene essentiality [15, 18, 20, 26]. Here, we explore a number of protein properties, obtained from mouse protein sequence data, to test their efficacy at distinguishing essential genes from viable genes in mouse.

#### Simple sequence features

**Fig 5:**
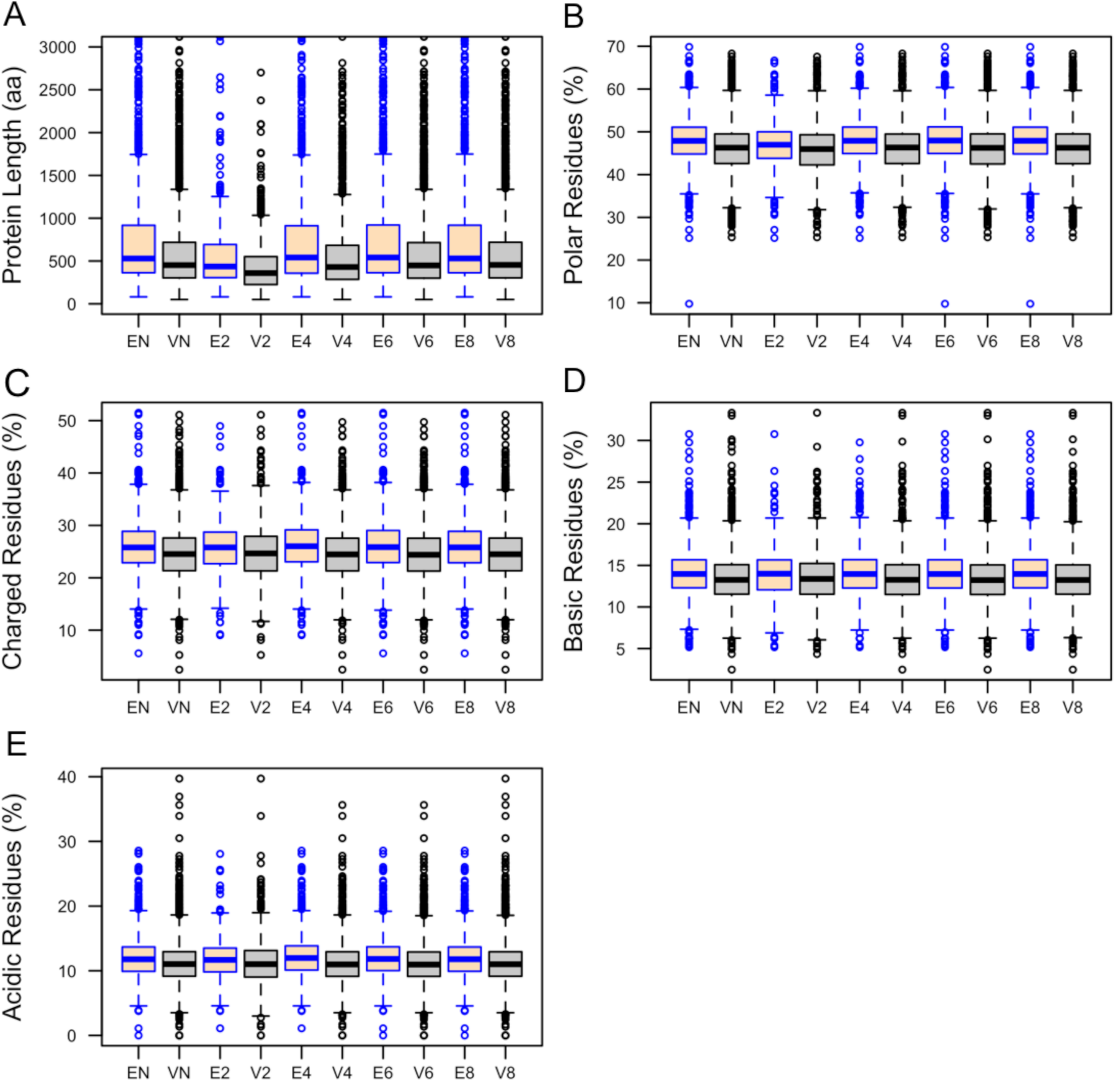
**Distributions of (A) length, (B) polar, (C) charged, (D) basic, and (E) acidic residues (%) of proteins encoded by essential and viable genes.** Here, EN and VN refer to essential and viable genes in the non–culled dataset. Ex and Vx define essential and viable genes in the culled dataset where all coded proteins share sequence similarity less than x0%. Top 2% longest proteins (A) were excluded from the datasets to make plots more readable. In this box plot, the top and bottom of the box denote the upper and lower quartiles; the line inside the box denotes the median; and individual points denote the outliers.

We found that essential proteins have significantly longer lengths than viable proteins (529aa versus 452aa (median length); p–value = 1.03×10^−21^ Mann-Whitney U test). The distributions of protein lengths between essential and viable proteins within the non-culled and culled datasets are variable and discriminate between these classes (Fig 5A). We also found variations in the frequencies of amino acids found in the proteins encoded by essential and viable genes (Table 5). Proteins encoded by essential genes in the non-culled dataset tend to have higher proportions of Ala, Asp, Glu, Lys, Gln and Ser. Distributions of Lys residues demonstrated the same trend for all culled datasets. Essential proteins in the 40%, 60% and 80% culled dataset also had more Asp, Glu and Gln compared to viable proteins (S3 Data). Viable proteins have more Leu, Cys, Phe, Val and Trp.

**Table 5:** Differences in the frequency of usage of the 20 amino acids between essential and viable mouse proteins in the non-culled dataset. The p-value for the Bonferroni correction is 0.0025.

Amino acid	Essential	Viable	p-value
**A**	6.87	6.74	1.1×10^−3^
**C**	1.88	2.08	1.9×10^−7^
**D**	4.91	4.73	4.1×10^−7^
**E**	6.68	6.22	6.5×10^−12^
**F**	3.39	3.80	1.8×10^−17^
**G**	6.43	6.49	0.28
**H**	2.48	2.39	5.8×10^−3^
**I**	4.06	4.24	3.2×10^−4^
**K**	5.67	5.15	3.6×10^−14^
**L**	9.31	10.00	2.7×10^−21^
**M**	2.19	2.21	0.84
**N**	3.63	3.50	2.9×10^−3^
**P**	5.86	5.72	0.026
**Q**	4.48	4.25	3.8×10^−7^
**R**	5.41	5.38	0.33
**S**	8.01	7.78	1.4×10^−3^
**T**	5.14	5.24	8.4×10^−3^
**V**	5.89	6.25	2.7×10^−12^
**W**	1.01	1.31	8.8×10^−24^
**Y**	2.73	2.83	0.019

**Fig 6:**
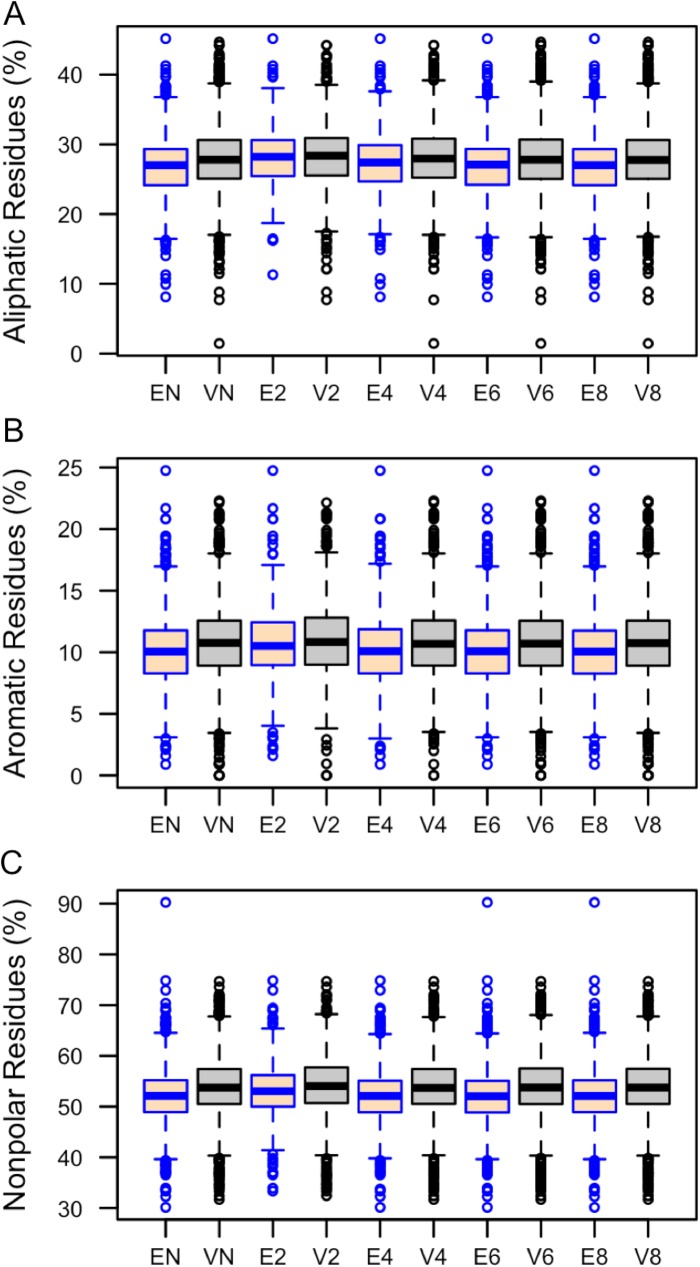
**Distributions of (A) aliphatic, (B) aromatic, and (C) non-polar residues (%) between essential and viable proteins.** Here, EN and VN refer to essential and viable genes in the non–culled dataset. Ex and Vx define essential and viable genes in the culled dataset where all coded proteins share sequence similarity less than x0%. In this box plot, the top and bottom of the box denote the upper and lower quartiles; the line inside the box denotes the median; and individual points denote the outliers.

Protein average molecular weight, charge, isoelectric point and frequencies of different amino acid categories were computed using the tool Pepstats [27]. Proteins encoded by essential genes have a significantly higher average molecular weight (MW) compared to proteins encoded by viable genes (Table 6). Differences for charge, isoelectric point, tiny and small residues were not statistically significant. Essential proteins were found to have greater proportions of polar (Fig 5B), charged (Fig 5C), basic (Fig 5D) and acidic (Fig 5E) amino acids. In contrast, proteins encoded by viable genes have significantly higher proportions of aliphatic (Fig 6A), aromatic (Fig 6B) and non-polar residues (Fig 6C). However, the Mann–Whitney U test showed that differences of aliphatic (p-value = 0.42) and aromatic (p-value = 0.19) residues between essential and viable proteins in the 20% culled datasets are not statistically significant (Table 6).

**Table 6:** Median values of different protein features obtained from Pepstats and the p-values of their distribution calculated using the Mann–Whitney U test. The p-value for the Bonferroni correction is 0.0038.

Datasets		Protein Sequence Features							
		Molecular weight (Da)	Aliphatic (%)	Aromatic (%)	Non-polar (%)	Polar (%)	Charged (%)	Basic (%)	Acidic (%)
**Non-culled**	Essential	59146	27.0	10.0	52.1	47.9	25.8	14.0	11.8
	Viable	50446	27.8	10.8	53.7	46.3	24.5	13.3	11.0
	**p-value**	3.4×10^−21^	3.5×10^−13^	3.7×10^−14^	4.4×10^−27^	4.6×10^−27^	2.3×10^−18^	2.2×10^−15^	1.8×10^−13^
**Culled (20%)**	Essential	48926	28.2	10.5	53.0	47.0	25.8	14.0	11.7
	Viable	40327	28.4	10.8	54.0	46.0	24.7	13.4	11.0
	**p-value**	4.1×10^−10^	0.4	0.2	1.0×10^−4^	1.0×10^−4^	7.6×10^−5^	4.9×10^−4^	6.4×10^−4^
**Culled (40%)**	Essential	60212	27.4	10.1	52.1	47.9	26.0	14.0	12.0
	Viable	48362	28.0	10.7	53.7	46.3	24.5	13.3	11.0
	**p-value**	3.2×10^−21^	6.0×10^−6^	4.1×10^−9^	1.6×10^−20^	1.6×10^−20^	3.7×10^−19^	5.6×10^−12^	3.4×10^−16^
**Culled (60%)**	Essential	60237	27.1	10.1	52.0	48.0	25.9	14.0	11.8
	Viable	50058	27.8	10.7	53.8	46.2	24.4	13.2	11.0
	**p-value**	1.4×10^−21^	3.4×10^−11^	2.4×10^−12^	4.9×10^−28^	5.1×10^−28^	1.9×10^−21^	1.9×10^−15^	4.6×10^−17^
**Culled (80%)**	Essential	59285	27.0	10.0	52.1	47.9	25.8	14.0	11.8
	Viable	50479	27.8	10.7	53.7	46.3	24.5	13.2	11.0
	**p-value**	1.2×10^−20^	1.2×10^−12^	1.4×10^−14^	1.0×10^−27^	1.1×10^−27^	3.9×10^−19^	8.6×10^−16^	3.4×10^−14^

#### Enzyme class

Almost all cellular processes are dependent on the presence of enzymes. Enzymatic function thereby could be another indicator of gene essentiality. We extracted the annotations of the six primary enzyme classes from UniProt [28] and counted the number of essential and viable proteins belonging to each of these classes (Table 7). In the non–culled datasets, 29.8% (388/1301) of the total number of essential proteins exhibit enzymatic activity compared to 27.7% (956/3451) of viable proteins, though this difference is not statistically significant. The culled datasets also show variations within each class in the percentage of proteins that function as enzymes (Table 7). Analysis of the enzyme classifications shows that the proteins encoded by essential genes are rich in transferases and ligases as compared to those encoded by viable genes. Hydrolases were found to be strongly associated with viable proteins in the non–culled dataset. No statistically significant differences between the datasets were observed for oxidoreductases, lyases and isomerases.

**Table 7:** Differences in the frequencies of different enzyme class observed between essential and viable mouse proteins. The Bonferroni corrected p-valu for the Chi-squared tests is 0.0083.

Datasets		Enzyme Classes					
		Oxidoreductase	Transferase	Hydrolase	Lyase	Isomerase	Ligase
**Non-culled**	Essential	3.4	13.5	8.1	1.31	0.69	2.9
	Viable	3.7	10.1	10.9	1.10	0.67	1.2
	**p-value**	0.59	1.81×10^−3^	5.88×10^−3^	0.56	0.92	5.43×10^−5^
**Culled (20%)**	Essential	6.05	12.94	10.02	2.92	1.46	2.92
	Viable	6.00	9.64	12.59	1.18	1.38	1.28
	**p-value**	0.97	0.068	0.18	0.017	0.90	0.027
**Culled (40%)**	Essential	3.95	13.42	9.26	1.77	0.94	3.23
	Viable	4.17	9.30	11.56	1.26	0.83	1.26
	**p-value**	0.781	9.2×10^−4^	0.070	0.26	0.75	1.6×10^−4^
**Culled (60%)**	Essential	3.46	13.83	8.07	1.40	0.74	3.13
	Viable	3.77	9.53	11.59	1.16	0.71	1.19
	**p-value**	0.63	1.1×10^−4^	1.4×10^−3^	0.52	0.91	1.4×10^−5^
**Culled (80%)**	Essential	3.41	13.56	7.90	1.32	0.70	2.94
	Viable	3.77	10.06	11.03	1.12	0.68	1.21
	**p-value**	0.56	1.3×10^−3^	2.7×10^−3^	0.58	0.94	4.5×10^−5^

#### Post-translational modifications and transcription

We investigated the frequency of annotations for four different post-translational modification keywords (‘phosphoprotein’, ‘glycoprotein’, ‘acetylation’ and ‘transcription’) as obtained from UniProt protein annotations. Protein phosphorylation plays crucial roles in regulating various cellular and metabolic processes, such as cell differentiation, cell division, survival etc. Around 30% of all eukaryotic proteins are estimated to be phosphorylated [29]. We found that essential proteins within the non-culled dataset are significantly more likely to be phosphorylated than viable proteins (51.42% versus 35.50%, p–value = 8.93×10^−15^). We observed the same trend for culled datasets (Table 8).

**Table 8:** Frequencies (%) of post-translational and transcription keywords in essential and viable mouse proteins and the corresponding p-values computed using the Chi-square test. The Bonferroni corrected p-value is 0.0125.

Datasets		Keywords			
		Phosphoprotein	Glycoprotein	Acetylation	Transcription
**Non-culled**	Essential	51.4	21.3	28.9	27.8
	Viable	35.5	38.2	12.9	11.5
	**p-value**	8.9×10^−15^	3.1×10^−19^	4.5×10^−33^	1.8×10^−36^
**Culled (20%)**	Essential	40.5	20.0	30.7	15.9
	Viable	29.2	33.2	16.3	7.9
	**p-value**	3.7×10^−4^	9.9×10^−6^	1.5×10^−8^	7.8×10^−6^
**Culled (40%)**	Essential	52.7	21.1	31.3	21.2
	Viable	32.7	38.1	13.6	9.6
	**p-value**	6.3×10^−17^	1.6×10^−14^	2.8×10^−26^	3.5×10^−17^
**Culled (60%)**	Essential	52.3	21.4	29.7	26.1
	Viable	34.6	38.8	12.7	11.1
	**p-value**	1.3×10^−16^	9.3×10^−19^	2.3×10^−33^	1.7×10^−29^
**Culled (80%)**	Essential	51.5	21.2	28.9	27.7
	Viable	35.4	38.4	12.6	11.5
	**p-value**	5.7×10^−15^	9.5×10^−20^	1.8×10^−33^	1.5×10^−35^

Greater than 50% of all proteins are glycosylated [30]. Glycoproteins are crucial for protein folding, solubility and localization [31]. A large number of them are secreted extracellular proteins, or are cell membrane proteins, and they therefore have roles in transport and cell–cell interactions. Viable protein are significantly more likely to be N-linked glycoproteins than essential proteins (Table 8).

Acetylated proteins in eukaryotes are those proteins that are post–translationally modified by the addition of an acetyl group at the N-terminus or on Lys side chains. The acetylation process is important for gene expression and metabolism. N-acetylated proteins also have vital roles in regulation of protein–protein interactions [32]. Proteins encoded by essential genes are more likely to have at least one acetyl group than proteins encoded by viable genes for all datasets (Table 8). Essential proteins in all datasets are thus more likely to be associated with regulating the transcription of genes, since acetylation is used to control gene expression.

#### Signal peptides

Signal peptides are short peptide sequences (usually 5–60 amino acids long) located at the N–terminus of a large number of newly synthesized proteins. They control the targeting and translocation of secreted or cell membrane proteins. Signal peptides direct proteins to different cellular locations (e.g. nucleus, mitochondria, endoplasmic reticulum, endosome, Golgi apparatus). Signal peptide motifs (computed using UniProt annotation and SignalP servers[33] are significantly more frequent in proteins encoded by viable genes as compared to proteins encoded by essential genes (Table 9).

**Table 9:** Signal peptide count in essential and viable proteins and the corresponding p–values computed using the Chi–square test.

Datasets	Essential	Viable	%Essential	%Viable	p-value
**Non-culled**	213	1004	16.4	29.1	1.2×10^−19^
**Culled (20%)**	67	304	14.0	29.9	8.3×10^−9^
**Culled (40%)**	151	698	15.7	30.3	8.8×10^−14^
**Culled (60%)**	200	941	16.5	30.3	1.8×10^−15^
**Culled (80%)**	210	993	16.3	29.3	4.1×10^−15^

#### Transmembrane domains

Transmembrane proteins extend through the lipid bilayer and span from the interior to the exterior of the cell. Transmembrane proteins usually adopt an α-helical structure while passing through the lipid bilayer once (single-pass proteins) or multiple times (multiple-pass proteins). Due to this structure, transmembrane proteins can mediate cellular functions both inside and outside of the cell. Transmembrane proteins are important for cell-cell communication, maintenance of cell structure, signalling, and ion transport. Many receptor proteins have a number of α-helical transmembrane domains spanning the cell membrane. Thus, the presence of transmembrane domains in protein encoded by essential and viable genes could be informative for functional annotation.

We found that the non-culled viable dataset is significantly enriched in transmembrane proteins (p-value = 1.86×10^−15^). Approximately 20% of essential proteins are annotated as transmembrane proteins, whereas the corresponding percentage is 34% for viable proteins. A total of 10.5% essential proteins consist of a single transmembrane helix, whereas this number is 17% for viable proteins. Also, 2% of essential proteins have seven transmembrane helices, compared to 6.5% of viable proteins. Overall, a greater number of viable proteins have transmembrane domains, and viable proteins have significantly more transmembrane helices per protein than essential transmembrane proteins.

### Gene ontology terms

Gene Ontology (GO) [34] is the most widely used scheme for classifying gene functions. The GO consortium provides a set of controlled vocabularies (ontology) to annotate the functional properties of gene and gene products across all species. Gene functions are annotated by means of three aspects: (a) molecular function (b) cellular component and (c) biological process. Here, we test whether GO term distributions vary between essential and viable genes.

#### Cellular component

Protein functions are closely related to the locations where they reside within a cell. Subcellular localisation has been shown to be important for predicting essential genes in prior studies [15, 18, 35]. As an example, eukaryotic proteins located in the nucleus carry out essential functions including DNA replication, mRNA synthesis and recombination. Subcellular localisation therefore should be useful in distinguishing mouse essential genes.

GO terms were extracted from the DAVID v6.8 functional annotation tool [36] by submitting the Ensembl IDs of mouse essential and viable genes. A total of 225 cellular component GO terms for essential genes and 149 terms for viable genes were retrieved, of which 53 and 82 terms were found significant, utilising the Bonferroni corrected p-value ≤ 0.05 from the functional annotation output of DAVID. Tables 10 and 11 summarise these cellular component GO terms favoured for essential and viable genes, respectively. Lists of the 50 most enriched GO terms for each class are listed in S4-S9. A majority of essential genes are intracellular. Terms most frequently associated with essential genes include: “nucleus”, “transcription factor complex”, “nucleoplasm”, “nucleolus”, and “intracellular membrane- bounded organelle”. Fifty-seven percent of total essential genes were found to be present in the nucleus.

**Table 10:** Top 20 enriched cellular component GO terms associated with essential mouse genes.

GO Term ID	GO Term Annotation	Count	%	Bonferroni Corrected p-Values
GO:0005634	nucleus	747	57.8	9.7x10^-95^
GO:0005667	transcription factor complex	104	8.04	1.4x10^-50^
GO:0005654	nucleoplasm	309	23.8	2.6x10^-50^
GO:0005737	cytoplasm	682	52.7	9.4x10^-46^
GO:0043234	protein complex	126	9.7	1.4x10^-27^
GO:0005829	cytosol	239	18.5	5.8x10^-22^
GO:0000790	nuclear chromatin	64	4.9	1.3x10^-20^
GO:0005925	focal adhesion	77	6.0	5.5x10^-15^
GO:0048471	perinuclear region of cytoplasm	103	8.0	4.9x10^-12^
GO:0009986	cell surface	97	7.5	5.4x10^-12^
GO:0005911	cell-cell junction	45	3.5	6.6x10^-10^
GO:0043025	neuronal cell body	80	6.2	4.9x10^-9^
GO:0043005	neuron projection	68	5.3	6.9x10^-9^
GO:0005730	nucleolus	107	8.3	8.3x10^-8^
GO:0030424	axon	60	4.6	1.1x10^-7^
GO:0000785	chromatin	31	2.4	5.0x10^-7^
GO:0030054	cell junction	90	7.0	2.0x10^-6^
GO:0005694	chromosome	54	4.2	2.7x10^-6^
GO:0043231	intracellular membrane-bounded organelle	93	7.2	2.9x10^-6^
GO:0017053	transcriptional repressor complex	20	1.5	3.4x10^-6^

**Table 11:** Top 20 enriched cellular component GO terms associated with viable mouse genes.

GO Term ID	GO Term Annotation	Count	%	Bonferroni Corrected p-Value
GO:0016020	membrane	1794	52.3	2.9x10^-111^
GO:0005886	plasma membrane	1298	37.8	2.1 x10^-79^
GO:0009986	cell surface	297	8.7	1.3 x10^-65^
GO:0005887	integral component of plasma membrane	426	12.4	8.6x10^-62^
GO:0043025	neuronal cell body	249	7.3	7.2 x10^-54^
GO:0005615	extracellular space	503	14.7	4.2 x10^-52^
GO:0005576	extracellular region	549	16.0	5.3 x10^-49^
GO:0005829	cytosol	575	16.8	1.1x10^-47^
GO:0009897	external side of plasma membrane	168	4.9	1.6x10^-43^
GO:0045202	synapse	221	6.4	5.5x10^-42^
GO:0030425	dendrite	206	6.0	1.2x10^-35^
GO:0030424	axon	171	5.0	1.3x10^-35^
GO:0045121	membrane raft	136	4.0	1.0x10^-33^
GO:0043005	neuron projection	182	5.3	2.9x10^-33^
GO:0005737	cytoplasm	1448	42.2	7.6x10^-30^
GO:0070062	extracellular exosome	692	20.2	8.9x10^-30^
GO:0016324	apical plasma membrane	145	4.2	1.3x10^-26^
GO:0030054	cell junction	245	7.1	2.4x10^-26^
GO:0048471	perinuclear region of cytoplasm	237	6.9	4.7x10^-24^
GO:0045211	postsynaptic membrane	108	3.1	8.6x10^-24^

In contrast, as shown by our analysis of transmembrane helices, many viable genes are membrane bound. Viable genes were enriched for cellular component terms including “membrane”, “plasma membrane”, “cell surface”, “extracellular region”, “extracellular space” and “lysosome”. A high percentage of essential (46%) and viable (41%) genes were also found with the annotation of cytoplasm. Notably, an individual protein can have more than one subcellular localisation annotation.

Subcellular locations were also analysed using the UniProt annotation and the WoLF PSORT tool [37]. Table 12 summarises the results of the UniProt analysis. We found that a significantly higher proportion of viable proteins are localised in plasma membrane (23%), membrane (15%) and extracellular region (14%), compared to essential proteins. A higher percentage of essential proteins are found within the nucleus (48%), as compared to viable proteins (23%). The same trend was also observed for culled datasets.

**Table 12:** Subcellular locations of all essential and viable mouse proteins as annotated in the UniProt database. p–values were computed using the Chi–square test. Here, the Bonferroni corrected p-value = 0.0041.

Cellular Components	Essential	Viable	%Essential	%Viable	p-value
Nucleus	627	815	48.2	23.6	8.37×10^−43^
Cytoplasm	433	1014	33.3	29.4	0.030
Plasma membrane	170	805	13.1	23.3	3.4×10^−12^
Membrane (excluding plasma)	117	545	9.0	15.8	2.2×10^−8^
Extracellular	95	504	7.3	14.6	2.6×10^−10^
Mitochondrion	67	145	5.1	4.2	0.17
Endoplasmic Reticulum (ER)	70	192	5.4	5.6	0.81
Golgi	62	150	4.8	4.3	0.54
Lysosome	10	80	0.8	2.3	5.4×10^−4^
Peroxisome	5	22	0.4	0.6	0.30
Cell Junction	78	199	6.0	5.8	0.77
Cell Projection	47	130	3.6	3.8	0.81

Subcellular location prediction results from WoLF PSORT are summarised in Table 13. While the absolute numbers are often different from UniProt, the trends in differences between essential and viable are similar. In this case, the most significant enrichment for subcellular localisation of essential proteins was the nucleus. We observed that 70% of total essential proteins are located in the nucleus compared to 49% of viable proteins. The analysis of WoLF PSORT prediction results further confirmed the tendency for viable genes to be membrane bound (36%) and extracellular (39%). Viable proteins were also enriched for localisation to endoplasmic reticulum (18%) and lysosome (11%) as compared to essential proteins.

**Table 13:** Subcellular locations of all essential and viable mouse proteins, which were predicted by WoLF PSORT. p–values were computed using the Chi–square test. Here, the Bonferroni corrected p-value = 0.0056.

Cellular Components	Essential	Viable	%Essential	%Viable	p-value
Nucleus	921	1712	70.8	49.6	2.2×10^−18^
Cytoplasm	700	1556	53.8	45.1	1.0×10^−4^
Plasma membrane	307	1261	23.6	36.5	4.3×10^−12^
Extracellular	353	1377	27.1	39.9	7.8×10^−11^
Mitochondrion	321	890	24.7	25.8	0.50
Endoplasmic Reticulum (ER)	183	621	14.1	18.0	3.3×10^−3^
Golgi	45	156	3.5	4.5	0.11
Lysosome	86	398	6.6	11.5	2.1×10^−6^
Peroxisome	204	623	15.7	18.1	0.08

These analyses of cellular localisations indicate that proteins encoded by essential genes are commonly located in the nucleus, whereas viable proteins are more likely to be extracellular or membrane bound. Viable proteins are also more likely to be located in the lysosome.

#### Biological processes

A total of 1,575 biological process terms were retrieved for essential genes, with 1,777 terms for viable genes, of which 323 terms for essential and 315 terms for viable datasets were significant meeting the Bonferroni corrected p-value ≤ 0.05. Table 14 lists the top 20 biological process terms significantly favoured for essential genes. Essential genes are often involved in developmental processes, as expected (Table 15). Significant enrichment for processes related to “transcription”, “cell proliferation”, “cell differentiation”, “organ morphogenesis”, “cell division”, and “DNA replication” is observed in the essential genes dataset. Biological process terms commonly annotated for viable genes include “inflammatory response”, “signal transduction”, “ion transport”, “immune response”, “response to drug”, “response to stimulus”, “behaviour”, “transmembrane transport”, “aging” and “regulation of apoptotic process” (Table 15).

**Table 14:** Top 20 enriched GO terms for essential mouse genes that are related to biological processes.

GO Term ID	GO Term Annotation	Count	%	Bonferroni Corrected p-Value
GO:0045944	positive regulation of transcription from RNA polymerase II promoter	294	22.7	3.3x10^-105^
GO:0001701	in utero embryonic development	156	12.1	7.2x10^-95^
GO:0045893	positive regulation of transcription, DNA-templated	202	15.6	6.9x10^-84^
GO:0006351	transcription, DNA-templated	365	28.2	3.4x10^-75^
GO:0000122	negative regulation of transcription from RNA polymerase II promoter	217	16.8	5.8x10^-75^
GO:0007507	heart development	130	10.1	5.3x10^-74^
GO:0007275	multicellular organism development	255	19.7	1.5x10^-71^
GO:0006355	regulation of transcription, DNA-templated	398	30.8	1.4x10^-69^
GO:0010628	positive regulation of gene expression	112	8.7	1.0x10^-33^
GO:0008284	positive regulation of cell proliferation	132	10.2	1.9x10^-33^
GO:0043066	negative regulation of apoptotic process	135	10.4	4.3x10^-33^
GO:0009887	organ morphogenesis	55	4.3	1.7x10^-29^
GO:0045892	negative regulation of transcription, DNA-templated	131	10.1	3.6x10^-29^
GO:0006357	regulation of transcription from RNA polymerase II promoter	105	8.1	7.8x10^-29^
GO:0009952	anterior/posterior pattern specification	55	4.3	1.0x10^-28^
GO:0001525	angiogenesis	78	6.0	4.6x10^-27^
GO:0008285	negative regulation of cell proliferation	100	7.7	1.3x10^-26^
GO:0003007	heart morphogenesis	41	3.2	1.5x10^-26^
GO:0001568	blood vessel development	42	3.2	2.6x10^-25^
GO:0001570	vasculogenesis	41	3.2	4.6x10^-25^

**Table 15:** Top 20 enriched GO terms for viable mouse genes that are related to biological processes.

GO Term ID	GO Term Annotation	Count	%	Bonferroni Corrected p-Value
GO:0006954	inflammatory response	208	6.1	1.2x10^-61^
GO:0002376	immune system process	219	6.4	3.8x10^-61^
GO:0007165	signal transduction	427	12.4	2.3x10^-38^
GO:0042493	response to drug	182	5.3	1.9x10^-37^
GO:0032496	response to lipopolysaccharide	124	3.6	3.5x10^-35^
GO:0043065	positive regulation of apoptotic process	158	4.6	8.8x10^-29^
GO:0045087	innate immune response	170	5.0	1.2x10^-24^
GO:0007204	positive regulation of cytosolic calcium ion concentration	90	2.6	1.3x10^-24^
GO:0045944	positive regulation of transcription from RNA polymerase II promoter	327	9.5	1.7x10^-24^
GO:0042981	regulation of apoptotic process	105	3.1	1.8x10^-23^
GO:0007568	aging	99	2.9	1.4x10^-22^
GO:0006955	immune response	141	4.1	5.5x10^-22^
GO:0006468	protein phosphorylation	212	6.2	8.2x10^-22^
GO:0006915	apoptotic process	207	6.0	2.8x10^-20^
GO:0019233	sensory perception of pain	56	1.6	3.4x10^-20^
GO:0007155	cell adhesion	183	5.3	8.5x10^-20^
GO:0006811	ion transport	207	6.0	2.5x10^-19^
GO:0045471	response to ethanol	73	2.1	1.6x10^-17^
GO:0006816	calcium ion transport	76	2.2	1.1x10^-16^
GO:0002250	adaptive immune response	74	2.2	3.2x10^-16^

#### Molecular function

Analysing the molecular function output generated by DAVID, a total of 265 terms for essential genes and 105 terms for viable genes were retrieved, of which 75 and 81 terms were significant, respectively (Tables 16 and 17). Essential genes are more likely to be annotated as being involved in “DNA binding”, “transcription factor activity”, “transcription factor binding”, and “transferase activity”. Viable genes are more likely to have the annotations of “signal transducer activity”, “ion channel activity”, “hydrolase activity”, “transporter activity”, “calcium ion binding”, “receptor binding”, “SH3 domain binding”, and “lipid binding”. A higher percentage of essential and viable genes were also found to be annotated as being involved in “protein binding”, “ATP binding”, “protein kinase binding”, and “protein kinase activity”.

**Table 16:** Top 20 enriched GO terms for essential mouse genes that are related to molecular function.

GO Term ID	GO Term Annotation	Count	%	Bonferroni Corrected p-Value
GO:0005515	protein binding	669	51.7	1.6x10^-121^
GO:0003677	DNA binding	356	27.5	3.2x10^-71^
GO:0043565	sequence-specific DNA binding	186	14.4	1.1x10^-62^
GO:0003700	transcription factor activity, sequence-specific DNA binding	206	15.9	4.4x10^-51^
GO:0003682	chromatin binding	131	10.1	8.0x10^-40^
GO:0008134	transcription factor binding	108	8.4	5.4x10^-37^
GO:0001077	transcriptional activator activity, RNA polymerase II core promoter proximal region sequence-specific binding	94	7.3	3.1x10^-36^
GO:0000978	RNA polymerase II core promoter proximal region sequence-specific DNA binding	108	8.4	2.3x10^-35^
GO:0044212	transcription regulatory region DNA binding	86	6.7	6.6x10^-35^
GO:0046982	protein heterodimerization activity	109	8.4	3.3x10^-21^
GO:0001228	transcriptional activator activity, RNA polymerase II transcription regulatory region sequence-specific binding	43	3.3	2.0x10^-18^
GO:0001085	RNA polymerase II transcription factor binding	31	2.4	5.4x10^-17^
GO:0019901	protein kinase binding	90	7.0	2.6x10^-16^
GO:0032403	protein complex binding	79	6.1	7.4x10^-16^
GO:0003705	transcription factor activity, RNA polymerase II distal enhancer sequence-specific binding	32	2.5	1.9x10^-15^
GO:0019899	enzyme binding	80	6.2	3.1x10^-14^
GO:0000979	RNA polymerase II core promoter sequence-specific DNA binding	31	2.4	3.6x10^-14^
GO:0042826	histone deacetylase binding	38	2.9	1.2x10^-12^
GO:0000977	RNA polymerase II regulatory region sequence-specific DNA binding	54	4.2	4.6x10^-12^
GO:0042803	protein homodimerization activity	121	9.4	9.8x10^-12^

**Table 17:** Top 20 enriched GO terms for viable mouse genes that are related to molecular function.

GO Term ID	GO Term Annotation	Count	%	Bonferroni Corrected p-Value
GO:0005515	protein binding	1242	36.2	3.3x10^-93^
GO:0004871	signal transducer activity	259	7.6	1.4x10^-33^
GO:0042803	protein homodimerization activity	293	8.5	1.7x10^-30^
GO:0005216	ion channel activity	97	2.8	1.2x10^-25^
GO:0005102	receptor binding	173	5.0	2.5x10^-24^
GO:0019901	protein kinase binding	170	5.0	2.7x10^-19^
GO:0004672	protein kinase activity	192	5.6	1.4x10^-18^
GO:0046982	protein heterodimerization activity	188	5.5	8.1x10^-18^
GO:0016301	kinase activity	221	6.4	9.6x10^-16^
GO:0005125	cytokine activity	95	2.8	2.8x10^-14^
GO:0042802	identical protein binding	215	6.3	3.1x10^-14^
GO:0043565	sequence-specific DNA binding	198	5.8	4.1x10^-11^
GO:0008083	growth factor activity	67	2.0	3.1x10^-10^
GO:0004872	receptor activity	75	2.2	5.4x10^-10^
GO:0002020	protease binding	58	1.7	6.4x10^-10^
GO:0019899	enzyme binding	134	3.9	9.1x10^-10^
GO:0008201	heparin binding	67	2.0	6.7x10^-9^
GO:0001077	transcriptional activator activity, RNA polymerase II core promoter proximal region sequence-specific binding	99	2.9	1.9x10^-8^
GO:0008144	drug binding	54	1.6	4.3x10^-8^
GO:0004896	cytokine receptor activity	30	0.9	4.7x10^-8^

### Protein domains

Protein domains are spatially distinct structural and/or functional units of a protein. They carry out particular functions or interactions, thereby contributing towards the overall functionality of a protein. We obtained domain data for essential and viable mouse proteins by analysing the functional annotation output of DAVID (Tables 18 and 19). We observed a total of 11 and 30 Pfam domains[38] that are significantly enriched in essential and viable proteins, respectively. Domains such as homeobox, helix-loop-helix DNA-binding domain, T-box, protein kinase domain, Zinc finger, and C4 type domain (many of which are found in transcription factors) showed enrichment in essential proteins. Domains including 7- transmembrane receptor, SH2, ion transport, Fibronectin type III domain (fn3), and SH3 (many of which are found in membrane proteins) were more frequently found in viable proteins. Although viable proteins were annotated with having protein kinase and zf-c4 domains, these domains were more frequently found within essential proteins.

**Table 18:** Key domains from the Pfam database that are enriched in proteins encoded by essential mouse genes.

Term ID	Term Annotation	Count	%	Bonferroni Corrected p-Value
PF00046	Homeobox domain	63	4.9	1.8x10^-17^
PF00010	Helix-loop-helix DNA-binding domain	28	2.2	4.7x10^-7^
PF07714	Protein tyrosine kinase	29	2.2	5.0x10^-5^
PF00110	Wnt family	11	0.9	8.7x10^-5^
PF00907	T-box	10	0.8	3.7x10^-4^
PF00008	EGF-like domain	19	1.5	7.1x10^-4^
PF00105	Zinc finger, C4 type (two domains)	14	1.1	7.6x10^-3^
PF00688	TGF-beta propeptide	10	0.8	8.7x10^-3^
PF00104	Ligand-binding domain of nuclear hormone receptor	14	1.1	0.012
PF00019	Transforming growth factor beta like domain	12	0.9	0.018
PF00069	Protein kinase domain	49	3.8	0.019

**Table 19:** Key domains from the Pfam database that are enriched in proteins encoded by viable mouse genes.

Term ID	Term Annotation	Count	%	Bonferroni Corrected p-Value
PF00001	7 transmembrane receptor (rhodopsin family)	162	4.7	4.2x10^-41^
PF00017	SH2 domain	56	1.6	2.2x10^-14^
PF07714	Protein tyrosine kinase	65	1.9	1.1x10^-12^
PF00520	Ion transport protein	53	1.5	2.6x10^-10^
PF00018	SH3 domain	49	1.4	2.4x10^-8^
PF00069	Protein kinase domain	121	3.5	4.8x10^-8^
PF00104	Ligand-binding domain of nuclear hormone receptor	28	0.8	4.1x10^-6^
PF13895	Immunoglobulin domain	32	0.9	6.5x10^-6^
PF00105	Zinc finger, C4 type (two domains)	26	0.8	4.2x10^-5^
PF10613	Ligated ion channel L-glutamate- and glycine-binding site	15	0.4	5.6x10^-5^
PF00060	Ligand-gated ion channel	15	0.4	5.6x10^-5^
PF01582	TIR domain	16	0.5	1.2x10^-4^
PF00211	Adenylate and Guanylate cyclase catalytic domain	16	0.5	1.2x10^-4^
PF00619	Caspase recruitment domain	17	0.5	1.9x10^-4^
PF02931	Neurotransmitter-gated ion-channel ligand binding domain	23	0.7	4.1x10^-4^
PF02932	Neurotransmitter-gated ion-channel transmembrane region	23	0.7	4.1x10^-4^
PF00229	TNF(Tumour Necrosis Factor) family	14	0.4	7.6x10^-4^
PF00020	TNFR/NGFR cysteine-rich region	15	0.4	1.2x10^-3^
PF00041	Fibronectin type III domain	49	1.4	1.3x10^-3^
PF00045	Hemopexin	16	0.5	1.7x10^-3^
PF00102	Protein-tyrosine phosphatase	21	0.6	2.2x10^-3^
PF00433	Protein kinase C terminal domain	18	0.5	2.3x10^-3^
PF00595	PDZ domain (Also known as DHR or GLGF)	45	1.3	5.2x10^-3^
PF00413	Matrixin	15	0.4	6.2x10^-3^
PF01471	Putative peptidoglycan binding domain	14	0.4	0.011
PF00005	ABC transporter	24	0.7	0.014
PF00019	Transforming growth factor beta like domain	19	0.6	0.019
PF00130	Phorbol esters/diacylglycerol binding domain (C1 domain)	24	0.7	0.021
PF00230	Major intrinsic protein	10	0.3	0.031
PF00664	ABC transporter transmembrane region	14	0.4	0.042

### Protein-protein interactions

Protein-protein interactions (PPI) are intrinsic to almost all biological processes. Since the majority of proteins interact with each other to expedite accurate functionality, knowledge about their interactions is crucial to understand the molecular mechanisms of cellular processes. A prior study found significant differences in PPI network properties between the essential and viable genes of *S*. *cerevisiae* and *E*. *coli* [39]. Network-based attributes were also found to be fundamental to elucidate proteins activities within the cell [21]. We therefore expected that the study of PPI networks could be an indicator of essentiality of mouse proteins.

Mouse protein-protein interaction data was obtained from the I2D database [40]. The PPI data was examined with the intention of learning whether essential PPI networks differ in their network properties from their viable counterparts. We analysed both known and predicted mouse PPIs to ensure high quality PPIs. Two PPI networks namely *Known* (**K**) and *Known-Predicted* (**KP**) were constructed from all mouse PPIs. After removing self and duplicate interactions, the network of proteins encoded by essential genes (essential-**K)** contained 3,988 protein nodes and 8,074 interactions; the network of proteins encoded by viable genes (viable-**K)** included 4,879 protein nodes and 9,624 interactions. The network essential-**KP** consisted of 12,001 nodes and 73,426 interactions, whereas the corresponding numbers are 11,686 and 75,040 for the viable-**KP** network. We computed 9 network properties for each essential and viable protein to recognise their importance in each of the PPI networks. 403 (30%) essential and 1,622 (47%) viable proteins has no PPI interactions in the network **K**. For **KP** network, these numbers were 61 (4.69%) and 371 (10.75%), respectively. Hence, essential proteins are more likely to participate in PPIs than viable proteins.

**Fig 7:**
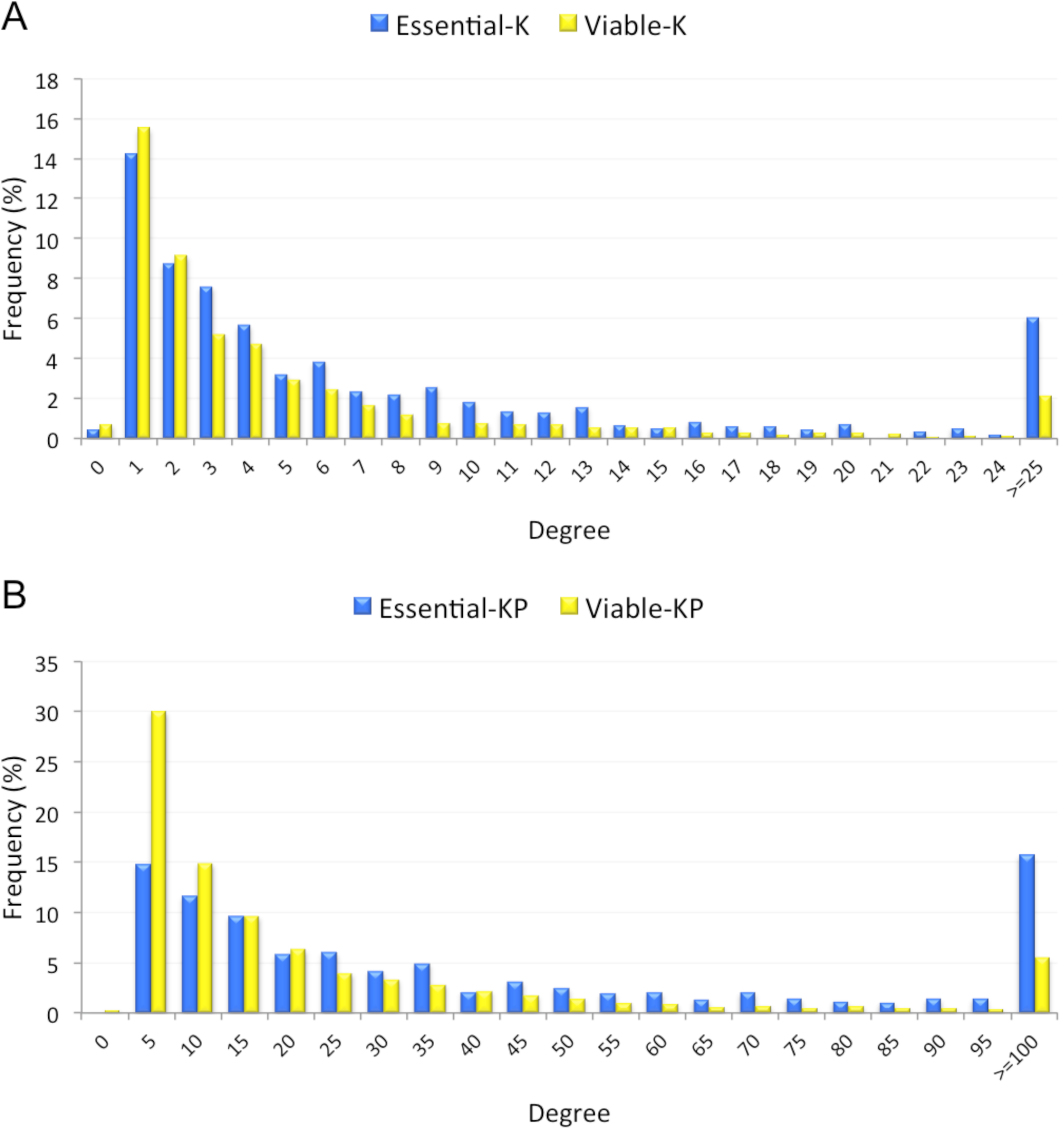
Degree distributions of essential and viable proteins involved in the *Known* (A) and *Known-Predicted* (B) protein-protein interaction (PPI) networks.

**Fig 8:**
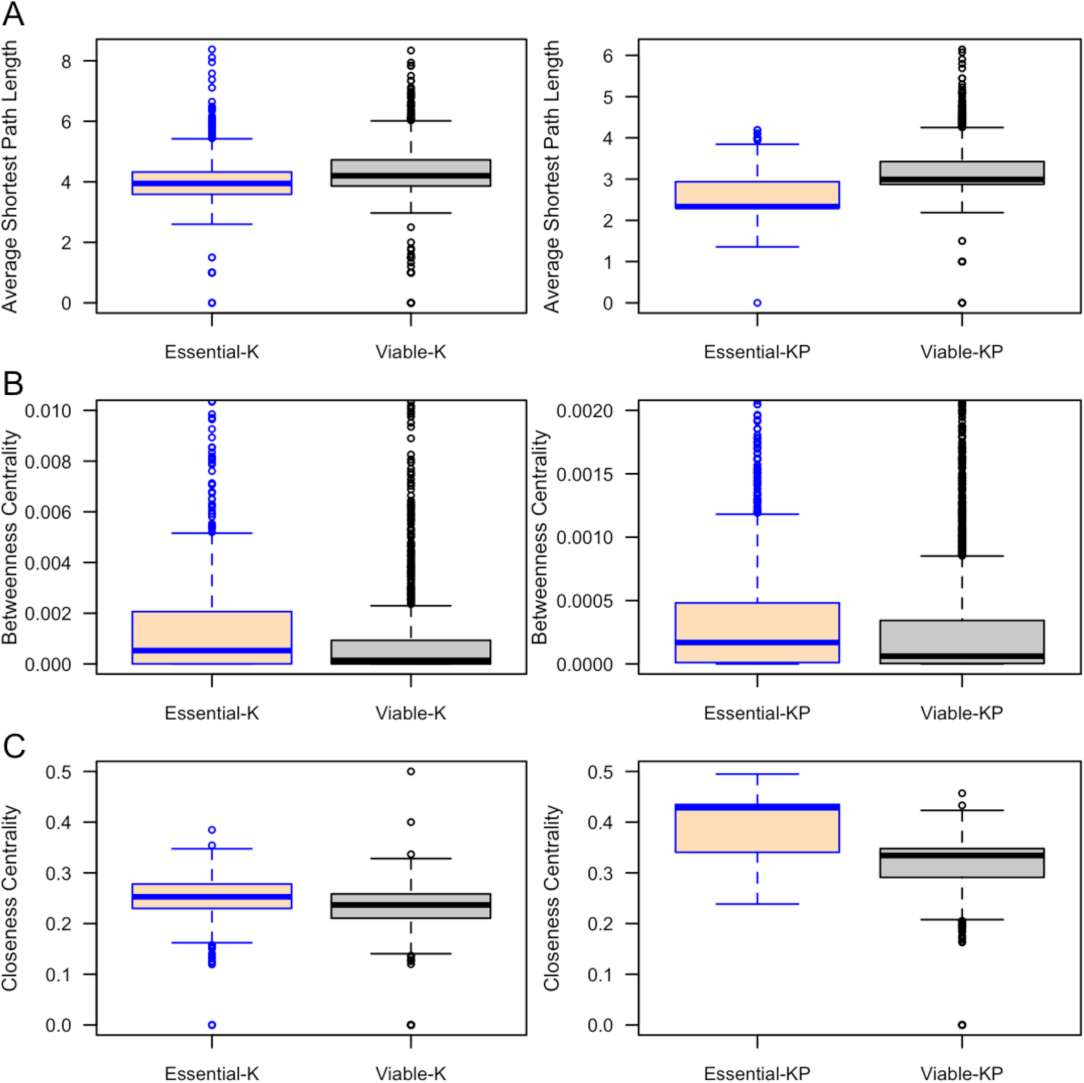
Length of ASP (A), Betweenness centrality (B) and Closeness centrality of essential and viable proteins in the *Known* and *Known-Predicted* PPI networks.

Our results demonstrated that essential proteins have more interactions (higher degrees) than viable proteins in both **K** and **KP** interaction networks (Fig 7, Table 20). The mean degree of essential proteins was higher than viable proteins for **K** (10.5 versus 6.4) and **KP** (57.7 versus 28.0). The Average Shortest Path (ASP) length is an indicator of a protein node’s efficiency in transporting information in a PPI network The ASP length of essential proteins is significantly shorter than the ASP length of viable proteins (Fig 8A, Table 20). The betweenness centrality is an indicator of the centrality of a protein node in the PPI network. The betweenness centrality of essential proteins in each of the interaction networks is significantly higher than that of viable proteins (Fig 8B, Table 20). We also found significantly higher clustering coefficient values for essential proteins in **K** and **KP** networks compared to viable proteins. Essential proteins tend to have significantly high closeness centrality than viable proteins (Fig 8C). This difference was statistically significant for both networks (Table 21).

**Table 20:** p-Values of the distributions of PPI network features between essential and viable datasets. p-values were computed using Mann–Whitney U test.

PPI Network Features	Known (K) Network	Known-Predicted (KP) Network
Degree	8.2×10^−16^	4.1×10^−63^
Average shortest path length	8.56×10^−26^	1.15×10^−260^
Betweenness centrality	1.89×10^−15^	3.21×10^−12^
Clustering coefficient	9.7×10^−4^	1.2×10^−39^
Closeness centrality	1.33×10^−28^	5.77×10^−266^

**Table 21:** Distributions of four network properties: BottleNeck (BN), Edge Percolation Component (EPC), Maximum Neighbourhood Component (MNC) and Density of Maximum Neighbourhood Component (DMNC) between essential and viable proteins. The Bonferroni corrected p-value in the Mann Whitney U test is 0.0125. Here, mean rank indicates which protein group holds higher values for a network property.

Network		Network Properties			
		BN	EPC	MNC	DMNC
**Known (*K***	Essential (Mean Rank	1522	1520	1486	1442
	Viable (Mean Rank	1287	1288	1304	1326
	**p-valu**	1.0×10^−17^	4.6×10^−13^	5.6×10^−10^	7.2×10^−5^
**Known-Predicted (*KP***	Essential (Mean Rank	2468	2683	2685	2147
	Viable (Mean Rank	2037	1950	1949	2166
	**p-valu**	9.1×10^−39^	3.1×10^−68^	2.4×10^−69^	0.641

**Fig 9:**
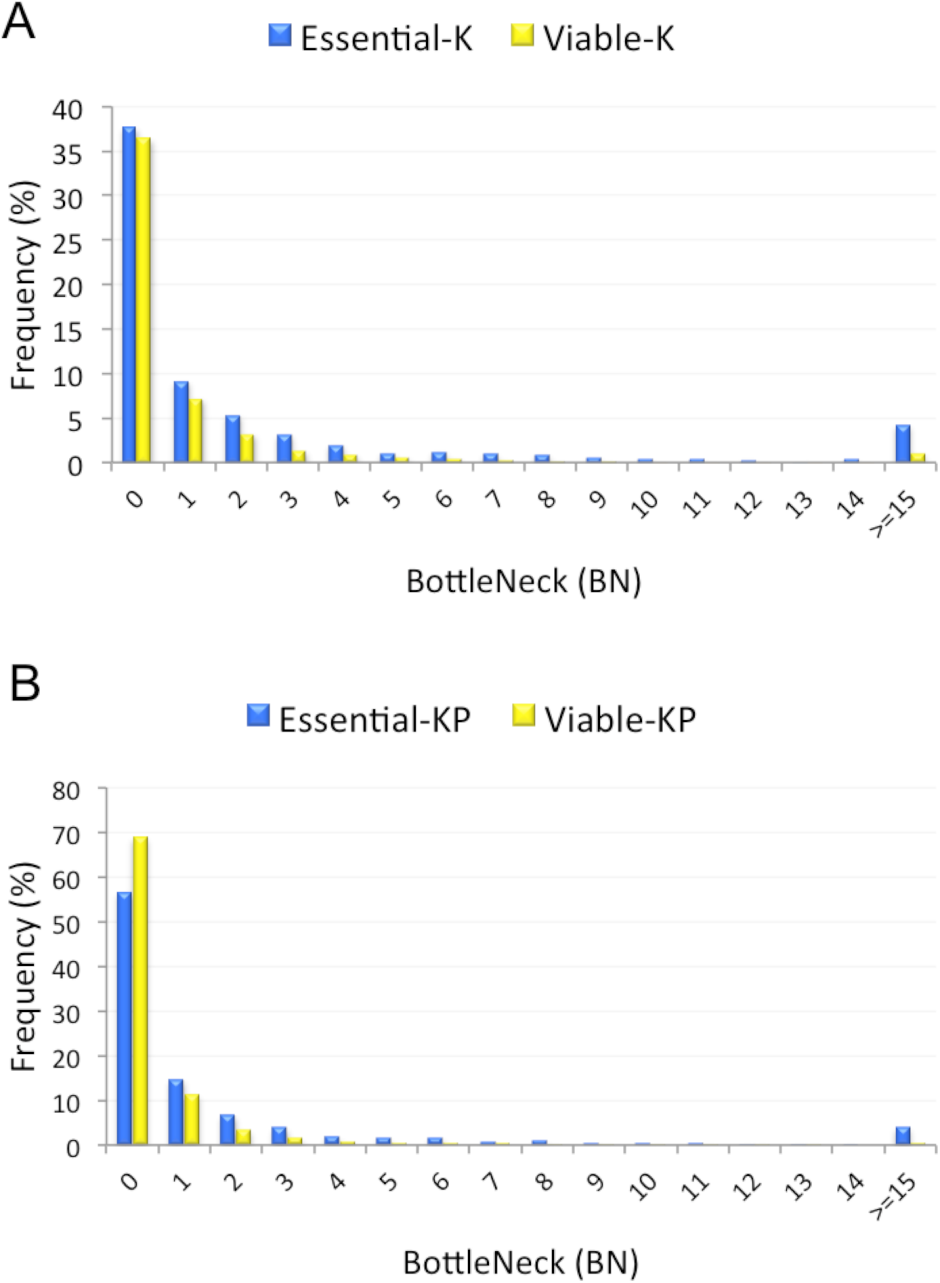
BottleNeck (BN) of essential and viable proteins in the *Known* (A) and *Known-Predicted* (B) protein-protein interaction (PPI) networks.

We wanted to identify protein nodes with large number of interactions (hubs) in the PPI network. We used the Hub object Analyser (Hubba) [41] to explore four additional network properties including: BottleNeck (BN), Edge Percolation Component (EPC), Maximum Neighbourhood Component (MNC) and Density of Maximum Neighbourhood Component (DMNC). These properties define probable hubs in the PPI network. Our investigation demonstrated that essential proteins tend to have high BN values in both **K** and **KP** networks (Fig 9). We further found that EPC and MNC of essential proteins are significantly higher than that of viable genes (Table 21). Although essential proteins exhibited high DMNC in the **K** network, the same trend was not observed for the **KP** network.

#### Housekeeping and enriched genes

Housekeeping genes are expressed at similar levels under all conditions, as they are required to maintain basic cellular functions [42]. In contrast, many genes are expressed only in certain conditions or environments, such as in individual tissues. We used the Pattern Gene Database [43] to test whether lethal and viable genes are also likely to be housekeeping or tissue-specific genes. Table 22 shows that viable genes are significantly more likely to be housekeeping or tissue enriched genes.

**Table 22:** Frequencies of housekeeping and tissue enriched genes.

	Essential	Viable	p-Value
**Housekeeping**	152 (11.7%)	562 (16.3%)	2.6 x 10^−4^
**Tissue Enriched**	445 (34.2%)	1511 (43.8%)	4.4 x 10^−6^

## Discussion

Understanding what makes a gene essential shows which cellular, developmental and tissue-specific processes are crucial for mammalian development. Our non-culled dataset contained a total of 1,301 essential and 3,451 viable mouse genes, which were obtained from the MGI database. We included only targeted deletion null mouse phenotypes in our analysis. A lengthy literature search corrected numerous inaccurate annotations within this database, giving accurate gene lists to analyse. The presence of multiple copies of similar proteins could bias the analysis; we thereby removed redundant proteins from our dataset to generate non-redundant or culled datasets. Comparing culled and non-culled datasets showed whether redundancy affects particular gene properties, though in general all sets follow similar trends.

We studied a wide range of gene and protein properties of *Mus musculus* genes, representative of different aspects of mouse biology, so that we could quantify their abilities to differentiate essential genes from viable genes. Our investigation focused on features that are attainable from existing databases and web-based tools. These properties fall into three categories: (1) genomic properties, which are based on gene sequence data. This group also included features such as evolutionary age and gene expression; (2) protein sequence properties, determined from protein sequence, including amino acid composition, enzyme class, post-translational modifications, signal peptides and transmembrane domains; (3) functional properties, which facilitate biological interpretations of gene functionality. These include GO annotations and PPIs. In total, we identified 75 features that show significant differences between essential and viable genes. These features, expressing different traits of mouse biology, are interrelated. Many (*e*.*g*. gene length, protein length, evolutionary age, gene expression, nuclear localization, PPI) are in broad agreement with those of previous studies on yeast [16–18] and bacteria [14, 15], but have not been verified in mammals. In addition to previously evidenced features, we found a number of important novel features that are strongly associated with essential genes, summarised in Table 23.

**Table 23:** Summary of characteristics likely to be associated with essential or viable genes.

Essential Gene Tendencies	Viable Gene Tendencies
More complex proteins, with greater length, more long introns and exons, and more transcripts	Simpler, shorter gene structure
Higher expression levels at all stages before juvenile	Lower expression levels at all stages before juvenile
Older evolutionary age	Younger evolutionary age
Transferase or ligase enzyme activity	Hydrolase enzyme activity
Phosphorylated and acetylated proteins	N- glycosylated proteins
Intracellular proteins	Secreted proteins
Nuclear proteins	Extracellular and membrane-bound proteins
Involved in developmental processes, such as: morphogenesis, proliferation, transcription, differentiation and cell division	Involved in extracellular interactions, such as: responses to stimuli, immune system, aging, signal transduction and transport
DNA binding functions	Transport functions
Central positions in protein-protein interaction networks	Peripheral positions in protein-protein interaction networks
Less likely to be housekeeping or tissue enriched genes	More likely to be housekeeping or tissue enriched genes

Mouse essential genes are more likely to be longer in length, and have more transcripts than viable genes. Essential genes also tend to exhibit more exons and have a longer exon length. These results are in agreement with a prior study which showed that longer genes with a large number of exons tend to exhibit a higher degree of alternative transcripts compared to smaller genes with fewer exons [44]. Essential genes thus tend to encode complex proteins, having multiple domains and diverse cellular or tissue specialisations [45]. Essential genes also tend to have a significantly longer length of introns and a lower GC content. Intron and exon length is known to vary inversely with GC content [46, 47]. GC content is also correlated with gene length [48] and recombination [49] in mammalian genomes.

Essential genes are expressed in greater proportions at the earlier stages of mouse development (pre-organogenesis stages) as compared to viable genes. Mouse genes that are expressed at early stages of development are more likely to be essential, as their disruption could affect downstream developmental events. We found that essential genes show a higher level of gene expression during development. This result is supported by previous studies that showed that highly expressed genes are likely to be essential and to evolve slowly [50, 51].

Essential genes tend to have older evolutionary origins than viable genes and thus are evolutionarily more conserved [5, 25], consistent with a previously reported observation that essential and highly expressed genes evolve more slowly than viable genes [52]. Overall, this analysis indicates that older mouse genes are more likely to be indispensible for fundamental cellular processes. Essential genes might be undergoing positive selection to retain their functionality, giving a lower mutation rate.

Numerous features derived from protein sequences differ between essential and viable gene products. We find that proteins encoded by essential genes tend to be longer in length and have greater molecular weight than proteins encoded by viable genes. This result is consistent with a prior study which stated that functionally essential proteins are more evolutionarily conserved and conserved proteins are, in general, longer in length [53]. Longer proteins contain more possible domains to mediate diverse cellular functionalities [45] and multiple protein–protein interactions, and our analysis with GO terms and protein domains supports this observation.

Essential proteins were found to have Ala, Asp, Glu, Lys, Gln and Ser residues in greater proportions. In contrast, viable proteins have higher proportions of Cys, Phe, Ile, Leu, Val and Trp. Cys can also form disulphide bonds, which are more common in extracellular proteins. A high Leu content is known to correlate negatively with the likelihood of being essential [20]. The enrichment of Lys in essential proteins agrees with our findings that they are likely to be more acetylated, as proteins are often acetylated on lysine residues [54]. The enrichment of acetylated proteins in essential datasets implies that they are involved in regulating protein-protein interactions, gene expression and metabolic processes [55].

Essential proteins are likely to have more polar, charged, basic and acidic amino acids, whereas viable proteins have more aliphatic, aromatic and non–polar residues, because viable proteins are more likely to be membrane proteins. Viable proteins also tend to have Src Homology 2 (SH2), Src Homology 3 (SH3) and ion transport domains. This further establishes the propensity of viable proteins being membrane–bound, as these evolutionary conserved protein domains are common constituents of membrane proteins. Signal peptide motifs are found to be more frequent in viable proteins, as they are more likely to be secreted.

Proteins encoded by essential genes are more likely to function as ligases or transferases, consistent with results from a recent study [14]. Our analysis of GO biological process annotations reveals that proteins encoded by essential genes are often involved in regulating DNA replication, DNA repair and transferase activity, consistent with these proteins functioning as ligases and transferases. The enrichment for hydrolases in viable datasets is logical, as hydrolases are less critical to cellular function. Ligases also perform more complex chemistry than hydrolases, which may indicate their essentiality.

Essential proteins are more likely to be phosphorylated, as phosphoproteins have critical roles in almost all cellular processes, including cell differentiation, gene transcription and cell division [56], confirmed by biological process GO terms. A greater number of N-glycosylated proteins in the viable datasets indicates the propensity of viable proteins to be membrane–bound or extracellular [57] and have a longer in vivo lifetime [58]. N-glycosylation can also aid folding of complex proteins [59]. Essential proteins are more likely to be engaged in control of gene transcription in the nucleus, as shown by GO and UniProt annotations and also by the presence of the zinc finger, C4 type (zf–C4) domain. Most occurrences of the zf–C4 domain are found within the DNA–binding regions of many nuclear receptors that function as transcription factors, which are enriched in the essential gene dataset.

Transmembrane proteins are common in the viable datasets, shown by various data, such as GO annotations, amino acid preferences and predicted number of transmembrane helices, consistent with their roles in cell communication, transport and signal transduction. These functions therefore appear to be less critical during mouse development.

Subcellular localisation has been recognised as a significant attribute for identifying essential genes in bacteria and yeast [15, 16, 18, 35]. We found that proteins encoded by mouse essential genes are more likely to be intracellular. The majority of these proteins are located in the nucleus. This is expected, because almost one third of eukaryotic nuclear proteins are encoded by essential genes and are responsible for carrying out vital cellular processes like DNA replication, DNA repair and transcription [60, 61]. Our analysis of GO biological process annotations further confirms this result. Proteins encoded by viable genes tend to be secreted (extracellular), as shown by the enrichment of signal peptide cleavage sites, fibronectin type III (fn3) domain and signal transducer activity. The fn3 protein domain is an evolutionarily conserved domain that is generally found in animal proteins, especially in extracellular proteins. Its main function is to mediate cell–cell signaling or interactions. These results agree with a recently reported study [14], which also showed the tendency of essential genes to encode nuclear proteins and viable genes to encode extracellular or membrane-bound proteins.

Unsurprisingly, GO data analysis shows that essential genes are more likely to be involved in developmental processes. These include the development of embryo, tissue, heart, nervous system, brain, lung, respiratory tube and blood vessel. Essential genes are enriched in T-box domains, which are vital for heart development [62]. We observed a significant enrichment of essential genes in cell morphogenesis, cell division, cell proliferation, DNA replication, cell differentiation, DNA repair and transcription, all crucial processes for growth. The presence of homeobox domains further confirms their vital role in morphogenesis. In contrast, viable genes were associated with inflammatory response, apoptosis, behaviour and immune response, which are processes unlikely to be required *in utero*. Unlike viable genes, essential genes tended to participate in activities such as protein binding, DNA binding, transcription factor binding, transcription, and ATP binding. Viable genes were linked to transport, ion channels, signal transduction (with SH2 protein domains), enzyme binding, receptor binding, and lipid binding, consistent with their location in membranes and involvement with communication with other cells. A recently published study by Dickinson et al [14], analysed GO terms for 410 essential and 1143 viable genes. Of these, only 89 essential and 297 viable genes are also present in our datasets. Dickinson et al. also found the tendency of essential genes to participate in cell differentiation, cell proliferation, transcription and DNA binding. Confirmation of a consistent set of GO annotations with a different gene dataset adds strong support to our conclusions regarding the functions of essential and viable genes. Viable genes are more likely to be housekeeping or tissue enriched.

The correlation between PPI networks and gene essentiality has already been established in bacteria [15, 16, 39], yeast [15, 35, 39, 63–65], fly [66] and human [21, 67]. Proteins that are highly connected in the PPI (hubs) tend to be essential and evolve slowly [68, 69], and their absence disrupts cell viability [70]. Shorter ASP length and high values of closeness centrality and clustering coefficient show that essential proteins can quickly transfer information to other reachable protein nodes in the PPI network.

We conclude that mammalian essential genes are significantly different from non–essential genes based upon a number of features. Our manually curated datasets allowed for a large number of statistically significantly differing features to be identified. The interdependency of various features implies that multiple aspects of biology unite to determine whether a gene is essential or non–essential in mammals. The features we have identified as strongly associated with mouse essential genes can be compared to characteristics of essential genes in other organisms to gain insights into the evolution of essential functions. The wide variety of features we have identified associated with essential and non-essential genes will allow for improvements in predicting whether a gene is essential. Due to the genome similarities between mice and humans, future analyses may facilitate the identification of human genetic disease candidates and potential therapeutic targets.

## Methods

### Datasets

To construct the datasets for the current research, the phenotype information of knockout mice was collected from the MGI database [22] (http://www.informatics.jax.org/phenotypes.shtml, accessed on 1 November 2013). We included only null alleles of mouse genes that have known phenotypes resulting from single gene targeted (knockout) deletions, because mutations generated by other methods may not have created complete loss of function alleles, and thus a viable hypomorphic allele could be associated with a gene that produces a lethal null allele. Genes were included in the essential dataset if they produced lethality in either the homozygous or heterozygous state on any strain background, and were not separated further within the dataset. The phenotype of a mouse gene was marked as essential or lethal if it is associated with any essentiality annotation in the MGI (including prenatal, perinatal and postnatal annotations). The term ‘prenatal essentiality’ is a valid Mammalian Phenotype Ontology term which is defined in MGI as death of the mice anytime between fertilization and birth, whereas, ‘perinatal essentiality’ is defined as death any time between embryonic day E18.5 and postnatal day 1. We used 18 phenotypic annotations to classify a single-gene knockout phenotype as nonessential or viable (S10 Data). Since the majority of these terms refer to processes or tissues present only after birth, homozygous knockouts of these genes are evidence of a viable phenotype. We manually checked the literature for phenotypes of knockouts of genes for viability that were linked to the “adipose tissue”, “abnormal skin morphology” and “abnormal skin physiology” terms, as these could be applied to embryos.

Our knockout datasets contained some ambiguous entries that have been annotated as both essential and viable in the MGI database. We manually checked phenotypes of these overlapped entries against the published literature and labelled them either as essential or viable. Each MGI gene symbol and identifier was further mapped to its corresponding Ensembl gene ID (http://www.ensembl.org) [71], UniGene expression clusters ID (http://www.ncbi.nlm.nih.gov/unigene) [24] and UniProt protein ID (http://www.uniprot.org/uniprot/) [28]. For some instances there were multiple UniProt protein IDs that correspond to one gene. For some of these cases, only one protein had the longest length and we included that in our dataset. For others, two or more protein IDs were found to have longest length. In these cases, to avoid bias due to annotation quality we included the longest length protein ID in our dataset that was marked as ‘reviewed’ in the UniProt annotations. Mouse protein sequences in FASTA format were downloaded from UniProt.

#### Non-redundant datasets

Redundancy was removed from our original essential and viable datasets by submitting the essential and viable protein sequences in FASTA format to Leaf [23] (http://leaf-protein-culling.appspot.com/). This gave four sets of non-redundant essential and viable proteins with protein pairs showing the maximum sequence similarities of 20%, 40%, 60% and 80% respectively. Protein sequences with <20% identity are structurally very different implying functional differences [72, 73] so we therefore did not generate non-redundant datasets by removing proteins with <20% sequence identities.

### Gene and protein sequence based features

We collected a number of gene and protein sequence based features to distinguish essential and viable phenotypes. Table 24 summarizes the sequence and functional attributes collected and the corresponding tools that were used to extract them.

**Table 24:** Sequence and functional features and corresponding bioinformatics tools.

Features	Bioinformatics Tools
**Genomic features:** gene length, % of GC content, number of transcripts, number of exons, length of exon and intron	Ensembl BioMart [74]
**Gene expression**	UniGene [24]
**Evolutionary age**	Ensembl gene trees [75]
**Protein sequence features:** protein length, molecular weight, protein charge, isoelectric point, amino acid composition	Pepstats [27]
**PPI network features**	I2D database (v2.3) [40], Cytoscape (v3.1.1) [76]
**Enzyme class**	UniProt [28]
**Keywords:** Glycoprotein, Phosphoprotein, Acetylation, Transcription	UniProt
**Transmembrane domains**	UniProt
**Subcellular localization**	UniProt, WoLF PSORT [37]
**Signal peptide**	SignalP 4.1 [33], UniProt
**Gene Ontology terms:** biological process, cellular component, molecular function	DAVID (v6.8) [77]
**Protein Domain**	DAVID (v6.8)

### Genomic properties

#### Gene sequence properties

Features including gene length (in base pair), % of GC content, number of transcripts, number of exons, lengths of exons and introns were retrieved from the Ensembl BioMart data mining tool [74] (http://www.ensembl.org/biomart/martview/) with the Ensembl release 75 dataset of the *Mus musculus* genes by submitting the Ensembl gene IDs. For genes with multiple transcripts, the longest length transcript was assessed. A gene’s exon number and the total exon length were calculated considering its longest transcript. The intron length of a gene was calculated by subtracting its total exon length from the corresponding gene length.

#### Gene expression

Raw expression data of mouse essential and viable genes were obtained from the NCBI UniGene database [24] as expressed sequence tag (EST) clusters using UniGene IDs. We retrieved EST clusters from 13 developmental stages: oocyte, unfertilized ovum, zygote, cleavage, morula, blastocyst, egg cylinder, gastrula, organogenesis, fetus, neonate, juvenile and adult. Since the total number of ESTs for a particular gene varies greatly between different developmental stages, we corrected the raw data to get gene expression in the form of transcripts per million (TPM). Eq 1 was used to estimate a TPM for the *i*^*th*^ gene at *j*^*th*^ developmental stage.

$$TPM_{i}^{j}=(Number\;of\;ESTs\;for\;i^{th}\;gene/Total\;ESTs\;in\;j^{th}\;stage)\times 10^{6}$$ (1)

#### Evolutionary age

Evolutionary ages of mouse protein coding genes were determined by analysing the Ensembl (release 75) gene trees [75]. These gene trees represent the evolutionary processes by which genes diverged from their common ancestors. Ensembl runs a orthology and paralogy gene prediction pipeline that uses the TreeBeST method from the TreeFam methodology [78] to generate rooted phylogenetic trees. This pipeline merges tree topologies with the corresponding species trees inferred from the NCBI taxonomy and generates Ensembl genes trees with the tree internal nodes being annotated for duplication or speciation events.

Gene evolutionary ages were extracted from these Ensembl genes trees. We assigned two evolutionary ages to a mouse gene of our datasets: the age of the MRD event and the age of the evolutionarily most distantly related species, *i*.*e*., the age of the DCA that has an identified homolog to that gene.

#### Protein sequence properties

We retrieved the length of our essential and viable protein sequences by querying the UniProtKB database with their UniProt IDs. A script in Python was developed to compute the percentage frequencies of each of the 20 amino acid residues within protein sequences.

Pepstats (http://emboss.bioinformatics.nl/cgibin/emboss/pepstats) is a EMBOSS suite program [27] which outputs a report comprising statistics of a number of properties about a FASTA formatted protein sequence. These attributes include: molecular weight, number of residues, charge, isoelectric point, and amino acid composition. This program groups amino acids into nine categories: Tiny (A, C, G, S and T); Small (A, B, C, D, G, N, P, S, T and V); Aliphatic (I, L and V); Aromatic (F, H, W and Y); Non-polar (A, C, F, G, I, L, M, P, V, W and Y); Polar (D, E, H, K, N, Q, R, S, T and Z); Charged (B, D, E, H, K, R and Z); Basic (H, K and R) and Acidic (B, D, E, Z). We used Pepstats to evaluate these sequence properties for our essential and viable protein sequences. The program was run with the default parameters setting. A Python script was written to extract features values from the output file generated by Pepstats.

#### Enzyme class

Primary EC numbers of mouse proteins were obtained from the definition lines (DE) of UniProtKB annotations by submitting UniProt IDs.

#### Post-translational modifications

Three post-translational modification (PTM) keywords ‘Glycoprotein’, ‘Phosphoprotein’ and ‘Acetylation’ were collected from the UniProtKB database for each protein of our datasets. The UniProt annotation ‘Glycoprotein’ is used for N–glycosylation sites.

We also collected information about the keyword ‘Transcription’. It is a keyword in the biological process category representing proteins involved in regulating the process of transcription.

#### Signal peptides

Protein signal peptides were predicted using the SignalP program v4.1 (http://www.cbs.dtu.dk/services/SignaP/) [33]. This program uses artificial neural network (ANN) and hidden Markov model (HMM) algorithms to predict the amino acid composition and the cleavage site position of the signal peptide. A script in Python was written to extract the HMM probabilities generated by SignalP which is considered as the measure for signal peptide prediction.

#### Transmembrane domains

We extracted the total number of transmembrane domains in each mouse protein by querying the UniProtKB database. Transmembrane helices are annotated in the UniProt feature table line (FT) as TRANSMEM. UniProt also outputs the information about the transmembrane domain locations in a protein sequence.

#### Subcellular location

Protein subcellular localizations were predicted from sequence data using the WoLF PSORT program (http://wolfpsort.org/) [37], chosen as it can make prediction on any protein sequence. WoLF PSORT predicts subcellular locations on the basis of known sorting signals, functional motifs and sequence features, such as amino acid composition. It outputs a report covering predicted locations with different confidence levels. We found prediction scores for six subcellular locations: nucleus, cytosol, plasma membrane, mitochondria, Golgi apparatus, peroxisome, and extracellular. We assigned a score of zero to a subcellular location if no prediction is made. We further collected information about all these six subcellular locations from the UniProtKB database. This feature is annotated as SUBCELLULAR LOCATION in the UniProt data file and is found in the comment lines (CC). The value of a subcellular location was set to 1 if found; otherwise, it was set to 0.

### Gene ontology terms

GO terms were obtained by using the ‘Functional Annotation’ tool of the web based application DAVID v6.8 (https://david.ncifcrf.gov/home.jsp) [36]. It integrates gene functional annotations with intuitive graphical displays to facilitate biological interpretations of any list of genes encoded by human, rat, mouse, or fly genomes. This program systematically associates a query gene list to their corresponding GO terms and highlights only the most pertinent terms among all along with their statistics. We extracted all possible GO terms for which the statistical test supported in DAVID has a p-value ≤0.05.

### Protein-protein interactions

Mouse PPI data was downloaded from the Interologous Interaction Database (I2D) v2.3 [40] which is an integrated repository of known, experimental and predicted PPIs for human, mouse, rat, fly, yeast and worm genomes. To obtain high quality PPI data, we analysed all known and predicted mouse PPIs. The data obtained from the I2D database were imported into Cytoscape (v3.1.1) [76] to visualize and analyze PPI network as a graph. In this case, we removed all self-loops and duplicate edges. The ‘network analyser’ plugin of Cytoscape was further used to determine network properties including degree, the length of average shortest path, betweenness centrality, clustering coefficient, and closeness centrality. We further determined four other network properties including BN, EPC, MNC and DMNC by using a web-based service called Hub Object Analyzer (Hubba) (http://hub.iis.sinica.edu.tw/Hubba/) [41]. This system deciphers and visualizes hubs from the user-provided PPI networks. Query proteins are ranked in Hubba based on their topological features. Hubba also generates a subgraph for the top *n* ranked (*n* ≤ 100) hub along with their identifier.

PPI networks are usually characterized as undirected graphs. As an example, let *G = (V*, *E)* be an undirected graph representing a PPI network. In the graph *G*, nodes *V* represent proteins and edges *E* = {(*a*,*b*) | *a*,*b* ∈ *V*} correspond to observed interactions between protein *a* and protein *b*. Graph topological feature are defined as:

#### Degree

The most elementary property of a protein *a* is its degree or connectivity, which is the number of interactions *a* has to the other proteins in the network.

#### Average shortest path length (ASP)

The shortest path measures the path with the minimum number of edges between proteins *a* and *b*. The ASP length therefore refers to the average over all shortest path length between all protein pairs.

#### Betweenness centrality (BC)

The betweenness centrality (BC) of a protein node *a* corresponds to the ratio of shortest paths passing through *a*[79, 80] and is computed as follows:

$$BC\left(a\right)=\underset{b\neq c\neq a\in V}{\sum }\frac{\sigma_{bc}\left(a\right)}{\sigma_{bc}}$$ (2)

Here, σ_bc_ denotes the number of shortest paths between proteins *b* and *c*; and σ_bc_(*a*) denotes the number of shortest paths between *b* and *c* to that go through protein node *a*.

#### Clustering coefficient (CCo)

The clustering coefficient (CCo) of protein *a* (Eq 3) measures the ratio of the number of connections between all nodes within the neighborhood of *a* to the maximum number connections that could possibly present between them [81]

$$CCo\left(a\right)=\frac{2e_{bc}}{\left(k_{a}\left(k_{a}-1\right)\right)}$$ (3)

Here, *e*_*bc*_ denotes the number of connections between all neighbors *b* and *c* of *a*, and *k*_*a*_ denotes the degree of *a*.

#### Closeness centrality (CC)

The closeness centrality (CC) of the protein *a* corresponds to the reciprocal of the sum of average shortest path length between *a* and all the other nodes within the network [82] (Eq 4). It measures how close a protein node is to all the other nodes in the PPI network.

$$CC\left(a\right)=\;\frac{1}{\sum_{b\neq a}d\left(a,\;b\right)}$$ (4)

Here, *d(a*, *b)* is the length of the average shortest path between proteins *a* and *b*.

BC, CCo and CC of each protein node are represented by a value between 0 and 1 where an isolated protein node has a value of 0 for these properties.

#### Bottleneck (BN)

Let, *T*_*r*_ be the shortest path tree derived from *G* considering protein node *r* ∈ *V* as the root node. Protein *b* ∈ *V* is a bottleneck node if at least n/4 nodes have their shortest path to *r* through *b* in *T*_*r*_. The BN score of the protein node *b* is defined to be the number of nodes *r* for which *b* is a bottleneck node in *T*_*r*_ [83].

#### Edge percolation component (EPC)

*G'* is a graph which is constructed *n* times from *G* by randomly removing a subset of edges. It is possible that proteins *a* and *b* are connected in *G* but not in *G'*. The EPC score [84] of the protein node *a* is computed using the following equation.

$$EPC\left(a\right)=\underset{b\neq a\in V}{\sum }\frac{\sum_{for\;each\;G^{\prime }}\{\begin{array}{c} a\;and\;b\;are\;connected\;in\;G^{\prime }\;1\\ else\;0 \end{array}}{n}$$ (5)

#### Maximum neighborhood component (MNC)

The MNC of a protein *a* refers to the size of the maximum connected component of the subnetwork induced by the neighborhood of *a* [41].

#### Density of maximum neighborhood component (DMNC)

The DMNC for the protein *a* is calculated using the following equation:

$$DMNC\left(a\right)=\frac{E_{M}}{N^{e}}$$ (6)

Here, *E*_*M*_ denotes the number of edges and *N* denotes the number of protein nodes of *DMNC(a); e* is a constant which is equal to 1.7.

#### Housekeeping and tissue specific genes

Assignments of genes as housekeeping or tissue specific were taken from the Pattern Gene Database (http://bioinf.xmu.edu.cn/PaGenBase/index.jsp) [43].

### Statistical analysis

Statistical tests were carried out throughout using the statistics software package SPSS v20. First, the normality of different features was assessed, as many significant statistical tests including parametric tests depend upon normal data. All sequence features were tested for normality using a one sample Kolmogorov–Smirnov Test (K–S test). If a sequence property showed a normal distribution, a two-sample t–test with unequal variance analysis was used. The t–test was applied to test the null hypothesis that two samples of independent observations come from identical normal distributions with equal means. Statistical significance was determined at the 0.05 level. The statistical significance of each property was determined using the two-tailed nonparametric Mann–Whitney U test when the samples did not show normal distribution. The Chi–squared (χ^2^) test was also carried out to check whether the frequencies of a particular feature in essential and viable gene differ from each other. We then applied the Bonferroni correction to calculate corrected p-values.

## Supporting information

S1 Data Mouse essential genes. (XLSX)

S2 Data Mouse viable genes. (XLSX)

S3 Data Differences in 20 amino acid frequencies observed between essential and viable mouse proteins in the culled datasets. (DOCX)

S4 Data Top 50 enriched cellular component GO terms associated with essential mouse genes. (DOCX)

S5 Data Top 50 enriched cellular component GO terms associated with viable mouse genes. (DOCX)

S6 Data Top 50 enriched GO terms for essential mouse genes that are related to biological processes. (DOCX)

S7 Data Top 50 enriched GO terms for viable mouse genes that are related to biological processes. (DOCX)

S8 Data Enriched GO terms for essential mouse genes that are related to molecular function. (DOCX)

S9 Data Top 50 enriched GO terms for viable mouse genes that are related to molecular function. (DOCX)

S10 Data Mammalian Phenotype (MP) annotations that were used for defining genes as either lethal or viable. (XLSX)

## Abbreviations

ASP: Average Shortest Path
BN: BottleNeck
CC: Closeness centrality
CCo: Clustering coefficient
DCA: Duplicate Common Ancestor
DMNC: Density of Maximum Neighbourhood Component
EPC: Edge Percolation Component
EST: Expressed Sequence Tag
GO: Gene Ontology
K: Known
KP: Known-Predicted
MNC: Maximum Neighbourhood Component
MGI: Mouse Genome Informatics
MRD: Most Recent Duplication
MW: Molecular Weight
PPI: Protein-Protein Interactions
PTM: Post-translational Modification
