# Supplementary material for: Properties of genes essential for mouse development
Source: PLoS One. 2017 May 31;12(5):e0178273. doi: 10.1371/journal.pone.0178273 (PMC5451031; doi:10.1371/journal.pone.0178273)
Supplement: S3 Data — (DOCX) [file pone.0178273.s003.docx]

**S3 Data.** **Differences in 20 amino acid frequencies observed between essential and viable mouse proteins in the culled datasets.** The p-value for the Bonferroni correction is 0.0025. L(xx) refers to essential and V(xx) refers to viable proteins in the culled dataset, where all coded proteins share sequence similarity less than xx%.

| **Amino acid** | **L(20)** | **V(20)** | **p-value** | **L(40)** | **V(40)** | **p-value** | **L(60)** | **V(60)** | **p-value** | **L(80)** | **V(80)** | **p-value** |
| --- | --- | --- | --- | --- | --- | --- | --- | --- | --- | --- | --- | --- |
| **A** | 6.91 | 6.82 | 0.40 | 6.88 | 6.74 | 6.1×10^-3^ | 6.82 | 6.74 | 0.010 | 6.87 | 6.74 | 1.1×10^-3^ |
| **C** | 1.96 | 2.07 | 0.03 | 1.86 | 2.1 | 1.7×10^-7^ | 1.86 | 2.11 | 2.2×10^-8^ | 1.86 | 2.08 | 8.1×10^-8^ |
| **D** | 4.86 | 4.71 | 0.03 | 4.98 | 4.7 | 1.9×10^-9^ | 4.93 | 4.69 | 1.4×10^-9^ | 4.92 | 4.72 | 1.4×10^-7^ |
| **E** | 6.65 | 6.31 | 4.0×10^-3^ | 6.84 | 6.21 | 6.8×10^-13^ | 6.71 | 6.2 | 1.7×10^-13^ | 6.68 | 6.21 | 3.3×10^-12^ |
| **F** | 3.8 | 3.94 | 0.13 | 3.47 | 3.79 | 5.6×10^-8^ | 3.41 | 3.77 | 2.3×10^-14^ | 3.39 | 3.79 | 9.2×10^-18^ |
| **G** | 6.28 | 6.31 | 0.15 | 6.21 | 6.35 | 0.09 | 6.36 | 6.47 | 0.13 | 6.42 | 6.5 | 0.22 |
| **H** | 2.33 | 2.33 | 0.74 | 2.44 | 2.4 | 0.68 | 2.47 | 2.4 | 0.036 | 2.48 | 2.39 | 7.5×10^-3^ |
| **I** | 4.51 | 4.18 | 0.03 | 4.2 | 4.2 | 0.43 | 4.1 | 4.18 | 0.038 | 4.06 | 4.23 | 3.5×10^-4^ |
| **K** | 5.8 | 5.36 | 6.9×10^-5^ | 5.81 | 5.15 | 7.9×10^-15^ | 5.69 | 5.09 | 9.9×10^-16^ | 5.67 | 5.13 | 2.6×10^-14^ |
| **L** | 9.82 | 10.4 | 1.5×10^-3^ | 9.52 | 10.2 | 3.2×10^-14^ | 9.33 | 10.1 | 5.5×10^-22^ | 9.32 | 10 | 7.8×10^-21^ |
| **M** | 2.31 | 2.35 | 0.88 | 2.22 | 2.23 | 0.59 | 2.18 | 2.19 | 0.63 | 2.18 | 2.21 | 0.99 |
| **N** | 3.57 | 3.48 | 0.16 | 3.64 | 3.51 | 0.01 | 3.64 | 3.5 | 1.6×10^-3^ | 3.63 | 3.5 | 4.8×10^-3^ |
| **P** | 5.29 | 5.42 | 0.37 | 5.63 | 5.66 | 0.71 | 5.83 | 5.74 | 0.19 | 5.88 | 5.73 | 0.020 |
| **Q** | 4.28 | 4.26 | 0.35 | 4.5 | 4.35 | 2.3×10^-3^ | 4.47 | 4.29 | 4.1×10^-5^ | 4.48 | 4.24 | 3.3×10^-7^ |
| **R** | 5.25 | 5.26 | 0.72 | 5.38 | 5.33 | 0.34 | 5.41 | 5.37 | 0.29 | 5.42 | 5.38 | 0.26 |
| **S** | 7.59 | 7.64 | 0.50 | 7.83 | 7.83 | 0.56 | 7.99 | 7.83 | 0.017 | 8.03 | 7.8 | 1.6×10^-3^ |
| **T** | 5.16 | 5.15 | 0.92 | 5.16 | 5.24 | 0.13 | 5.15 | 5.23 | 0.061 | 5.14 | 5.25 | 0.012 |
| **V** | 6.3 | 6.35 | 0.94 | 6.05 | 6.29 | 1.9×10^-4^ | 5.94 | 6.25 | 8.3×10^-9^ | 5.89 | 6.25 | 1.3×10^-11^ |
| **W** | 1.17 | 1.36 | 0.02 | 1.04 | 1.32 | 5.9×10^-14^ | 1.01 | 1.33 | 1.3×10^-23^ | 1.01 | 1.31 | 9.1×10^-25^ |
| **Y** | 3 | 2.84 | 0.35 | 2.72 | 2.81 | 0.08 | 2.74 | 2.82 | 0.122 | 2.73 | 2.83 | 0.024 |
