# Supplementary material for: Properties of genes essential for mouse development
Source: PLoS One. 2017 May 31;12(5):e0178273. doi: 10.1371/journal.pone.0178273 (PMC5451031; doi:10.1371/journal.pone.0178273)
Supplement: S4 Data — (DOCX) [file pone.0178273.s004.docx]

**S4 Data.** **Top 50 enriched cellular component GO terms associated with essential mouse genes.**

| **GO Term ID** | **GO Term Annotation** | **Count** | **%** | **Bonferroni Corrected p-Values** |
| --- | --- | --- | --- | --- |
| GO:0005634 | nucleus | 631 | 48.7 | 1.1x10^-76^ |
| GO:0044424 | intracellular part | 1005 | 77.6 | 3.3x10^-62^ |
| GO:0043227 | membrane-bounded organelle | 842 | 65.0 | 7.3x10^-62^ |
| GO:0043231 | intracellular membrane-bounded organelle | 841 | 64.9 | 1.3x10^-61^ |
| GO:0043229 | intracellular organelle | 903 | 69.7 | 5.8x10^-59^ |
| GO:0043226 | organelle | 903 | 69.7 | 7.8x10^-59^ |
| GO:0005622 | intracellular | 1017 | 78.5 | 2.0x10^-54^ |
| GO:0005654 | nucleoplasm | 151 | 11.7 | 7.2x10^-38^ |
| GO:0044428 | nuclear part | 226 | 17.5 | 6.8x10^-34^ |
| GO:0044451 | nucleoplasm part | 132 | 10.2 | 1.1x10^-33^ |
| GO:0031981 | nuclear lumen | 180 | 13.9 | 1.6x10^-32^ |
| GO:0043233 | organelle lumen | 202 | 15.6 | 2.5x10^-28^ |
| GO:0070013 | intracellular organelle lumen | 201 | 15.5 | 5.0x10^-28^ |
| GO:0031974 | membrane-enclosed lumen | 205 | 15.8 | 1.1x10^-27^ |
| GO:0005667 | transcription factor complex | 78 | 6.0 | 9.2x10^-27^ |
| GO:0044446 | intracellular organelle part | 396 | 30.6 | 2.0x10^-23^ |
| GO:0044422 | organelle part | 397 | 30.7 | 4.1x10^-23^ |
| GO:0043234 | protein complex | 282 | 21.8 | 8.8x10^-23^ |
| GO:0032991 | macromolecular complex | 309 | 23.9 | 1.5x10^-15^ |
| GO:0005694 | chromosome | 74 | 5.7 | 7.8x10^-11^ |
| GO:0044427 | chromosomal part | 62 | 4.8 | 1.1x10^-8^ |
| GO:0005737 | cytoplasm | 599 | 46.3 | 5.6x10^-7^ |
| GO:0031252 | cell leading edge | 30 | 2.3 | 2.1x10^-6^ |
| GO:0042995 | cell projection | 85 | 6.6 | 3.8x10^-6^ |
| GO:0016585 | chromatin remodeling complex | 18 | 1.4 | 4.7x10^-5^ |
| GO:0000785 | chromatin | 34 | 2.6 | 1.6x10^-4^ |
| GO:0070161 | anchoring junction | 28 | 2.2 | 2.8x10^-4^ |
| GO:0000792 | heterochromatin | 17 | 1.3 | 3.9x10^-4^ |
| GO:0043232 | intracellular non-membrane-bounded organelle | 204 | 15.8 | 4.1x10^-4^ |
| GO:0043228 | non-membrane-bounded organelle | 204 | 15.8 | 4.1x10^-4^ |
| GO:0000267 | cell fraction | 80 | 6.2 | 6.5x10^-4^ |
| GO:0000228 | nuclear chromosome | 27 | 2.1 | 7.0x10^-4^ |
| GO:0005819 | spindle | 25 | 1.9 | 0.001 |
| GO:0030027 | lamellipodium | 18 | 1.4 | 0.002 |
| GO:0005912 | adherens junction | 24 | 1.9 | 0.002 |
| GO:0005829 | cytosol | 73 | 5.6 | 0.003 |
| GO:0005626 | insoluble fraction | 70 | 5.4 | 0.005 |
| GO:0044454 | nuclear chromosome part | 23 | 1.8 | 0.005 |
| GO:0031594 | neuromuscular junction | 10 | 0.8 | 0.009 |
| GO:0005856 | cytoskeleton | 125 | 9.7 | 0.011 |
| GO:0000781 | chromosome, telomeric region | 9 | 0.7 | 0.014 |
| GO:0005624 | membrane fraction | 66 | 5.1 | 0.019 |
| GO:0000790 | nuclear chromatin | 15 | 1.2 | 0.027 |
| GO:0005913 | cell-cell adherens junction | 11 | 0.8 | 0.036 |
| GO:0015630 | microtubule cytoskeleton | 59 | 4.6 | 0.037 |
| GO:0000118 | histone deacetylase complex | 11 | 0.8 | 0.048 |
