# Supplementary material for: Properties of genes essential for mouse development
Source: PLoS One. 2017 May 31;12(5):e0178273. doi: 10.1371/journal.pone.0178273 (PMC5451031; doi:10.1371/journal.pone.0178273)
Supplement: S5 Data — (DOCX) [file pone.0178273.s005.docx]

**S5 Data.** **Top 50 enriched cellular component GO terms associated with viable mouse genes.**

| **GO Term ID** | **GO Term Annotation** | **Count** | **%** | **Bonferroni Corrected p-Value** |
| --- | --- | --- | --- | --- |
| GO:0005886 | plasma membrane | 1078 | 31.5 | 1.9x10^-122^ |
| GO:0044459 | plasma membrane part | 618 | 18.1 | 3.5x10^-66^ |
| GO:0009986 | cell surface | 168 | 4.9 | 9.4x10^-39^ |
| GO:0045202 | synapse | 169 | 4.9 | 4.5x10^-36^ |
| GO:0044421 | extracellular region part | 307 | 9.0 | 1.3x10^-34^ |
| GO:0005576 | extracellular region | 541 | 15.8 | 1.4x10^-32^ |
| GO:0009897 | external side of plasma membrane | 120 | 3.5 | 4.2x10^-30^ |
| GO:0005615 | extracellular space | 214 | 6.3 | 4.5x10^-27^ |
| GO:0044456 | synapse part | 118 | 3.5 | 4.6x10^-27^ |
| GO:0000267 | cell fraction | 232 | 6.8 | 4.1x10^-24^ |
| GO:0005626 | insoluble fraction | 209 | 6.1 | 1.4x10^-22^ |
| GO:0005624 | membrane fraction | 203 | 5.9 | 3.2x10^-22^ |
| GO:0030054 | cell junction | 186 | 5.4 | 7.5x10^-20^ |
| GO:0031226 | intrinsic to plasma membrane | 209 | 6.1 | 9.0x10^-20^ |
| GO:0043005 | neuron projection | 115 | 3.4 | 3.1x10^-18^ |
| GO:0005887 | integral to plasma membrane | 198 | 5.8 | 9.5x10^-18^ |
| GO:0045211 | postsynaptic membrane | 73 | 2.1 | 2.7x10^-17^ |
| GO:0042995 | cell projection | 200 | 5.9 | 6.8x10^-14^ |
| GO:0016020 | membrane | 1712 | 50.1 | 5.7x10^-12^ |
| GO:0043235 | receptor complex | 48 | 1.4 | 1.5x10^-10^ |
| GO:0030425 | dendrite | 57 | 1.7 | 3.5x10^-10^ |
| GO:0031225 | anchored to membrane | 85 | 2.5 | 6.4x10^-9^ |
| GO:0045177 | apical part of cell | 63 | 1.8 | 1.0x10^-8^ |
| GO:0005578 | proteinaceous extracellular matrix | 110 | 3.2 | 1.1x10^-8^ |
| GO:0031012 | extracellular matrix | 113 | 3.3 | 1.5x10^-8^ |
| GO:0042734 | presynaptic membrane | 23 | 0.7 | 3.0x10^-8^ |
| GO:0043025 | cell soma | 56 | 1.6 | 3.2x10^-8^ |
| GO:0016324 | apical plasma membrane | 50 | 1.5 | 3.6x10^-8^ |
| GO:0045121 | membrane raft | 45 | 1.3 | 5.1x10^-8^ |
| GO:0031982 | vesicle | 166 | 4.9 | 7.8x10^-8^ |
| GO:0044425 | membrane part | 1559 | 45.6 | 2.0x10^-7^ |
| GO:0031410 | cytoplasmic vesicle | 160 | 4.7 | 6.1x10^-7^ |
| GO:0030424 | axon | 48 | 1.4 | 1.2x10^-5^ |
| GO:0016323 | basolateral plasma membrane | 58 | 1.7 | 1.5x10^-5^ |
| GO:0031988 | membrane-bounded vesicle | 130 | 3.8 | 7.8x10^-5^ |
| GO:0005764 | lysosome | 66 | 1.9 | 1.6x10^-4^ |
| GO:0016023 | cytoplasmic membrane-bounded vesicle | 127 | 3.7 | 1.9x10^-4^ |
| GO:0000323 | lytic vacuole | 66 | 1.9 | 2.0x10^-4^ |
| GO:0014069 | postsynaptic density | 27 | 0.8 | 5.4x10^-4^ |
| GO:0008021 | synaptic vesicle | 32 | 0.9 | 0.001 |
| GO:0019717 | synaptosome | 31 | 0.9 | 0.002 |
| GO:0008328 | ionotropic glutamate receptor complex | 12 | 0.4 | 0.003 |
| GO:0034702 | ion channel complex | 57 | 1.7 | 0.003 |
| GO:0005829 | cytosol | 154 | 4.5 | 0.004 |
| GO:0005773 | vacuole | 69 | 2.0 | 0.004 |
| GO:0005737 | cytoplasm | 1412 | 41.3 | 0.004 |
| GO:0032994 | protein-lipid complex | 17 | 0.5 | 0.006 |
| GO:0043197 | dendritic spine | 17 | 0.5 | 0.006 |
| GO:0034358 | plasma lipoprotein particle | 17 | 0.5 | 0.006 |
| GO:0030136 | clathrin-coated vesicle | 41 | 1.2 | 0.007 |
| GO:0044463 | cell projection part | 58 | 1.7 | 0.017 |
| GO:0031224 | intrinsic to membrane | 1287 | 37.7 | 0.033 |
| GO:0033267 | axon part | 15 | 0.4 | 0.049 |
