# Supplementary material for: Properties of genes essential for mouse development
Source: PLoS One. 2017 May 31;12(5):e0178273. doi: 10.1371/journal.pone.0178273 (PMC5451031; doi:10.1371/journal.pone.0178273)
Supplement: S6 Data — (DOCX) [file pone.0178273.s006.docx]

**S6 Data.** **Top 50 enriched GO terms for essential mouse genes that are related to biological processes.**

| **GO Term ID** | **GO Term Annotation** | **Count** | **%** | **Bonferroni Corrected p-value** |
| --- | --- | --- | --- | --- |
| GO:0007275 | multicellular organismal development | 664 | 51.3 | 1.8x10^-203^ |
| GO:0032502 | developmental process | 687 | 53.1 | 5.3x10^-199^ |
| GO:0048856 | anatomical structure development | 596 | 46.0 | 9.3x10^-186^ |
| GO:0009790 | embryonic development | 333 | 25.7 | 4.6x10^-181^ |
| GO:0048513 | organ development | 508 | 39.2 | 1.3x10^-173^ |
| GO:0048731 | system development | 554 | 42.8 | 1.7x10^-167^ |
| GO:0009653 | anatomical structure morphogenesis | 387 | 29.9 | 3.2x10^-146^ |
| GO:0009792 | embryonic development ending in birth or egg hatching | 231 | 17.8 | 3.5x10^-132^ |
| GO:0043009 | chordate embryonic development | 229 | 17.7 | 4.9x10^-131^ |
| GO:0009888 | tissue development | 249 | 19.2 | 2.5x10^-101^ |
| GO:0032501 | multicellular organismal process | 715 | 55.2 | 1.1x10^-97^ |
| GO:0001701 | in utero embryonic development | 153 | 11.8 | 2.8x10^-89^ |
| GO:0009887 | organ morphogenesis | 227 | 17.5 | 3.0x10^-89^ |
| GO:0048598 | embryonic morphogenesis | 173 | 13.4 | 1.6x10^-85^ |
| GO:0048869 | cellular developmental process | 384 | 29.7 | 1.6x10^-83^ |
| GO:0007507 | heart development | 134 | 10.3 | 3.8x10^-81^ |
| GO:0030154 | cell differentiation | 369 | 28.5 | 8.4x10^-80^ |
| GO:0048646 | anatomical structure formation involved in morphogenesis | 168 | 13.0 | 6.2x10^-79^ |
| GO:0048519 | negative regulation of biological process | 325 | 25.1 | 2.4x10^-73^ |
| GO:0009987 | cellular process | 1065 | 82.2 | 4.2x10^-72^ |
| GO:0007389 | pattern specification process | 142 | 11.0 | 8.8x10^-72^ |
| GO:0048523 | negative regulation of cellular process | 302 | 23.3 | 6.3x10^-71^ |
| GO:0007399 | nervous system development | 247 | 19.1 | 1.3x10^-68^ |
| GO:0006357 | regulation of transcription from RNA polymerase II promoter | 206 | 15.9 | 4.5x10^-68^ |
| GO:0001944 | vasculature development | 128 | 9.9 | 9.1x10^-66^ |
| GO:0031323 | regulation of cellular metabolic process | 492 | 38.0 | 2.0x10^-65^ |
| GO:0035295 | tube development | 131 | 10.1 | 2.8x10^-65^ |
| GO:0019222 | regulation of metabolic process | 508 | 39.2 | 8.4x10^-65^ |
| GO:0001568 | blood vessel development | 125 | 9.7 | 3.8x10^-64^ |
| GO:0007417 | central nervous system development | 153 | 11.8 | 1.5x10^-63^ |
| GO:0048522 | positive regulation of cellular process | 305 | 23.6 | 1.5x10^-61^ |
| GO:0080090 | regulation of primary metabolic process | 469 | 36.2 | 2.2x10^-60^ |
| GO:0048568 | embryonic organ development | 120 | 9.3 | 1.4x10^-59^ |
| GO:0010468 | regulation of gene expression | 438 | 33.8 | 1.0x10^-58^ |
| GO:0045449 | regulation of transcription | 412 | 31.8 | 1.8x10^-57^ |
| GO:0048518 | positive regulation of biological process | 321 | 24.8 | 2.2x10^-57^ |
| GO:0051254 | positive regulation of RNA metabolic process | 155 | 12.0 | 2.2x10^-56^ |
| GO:0048729 | tissue morphogenesis | 116 | 9.0 | 3.7x10^-56^ |
| GO:0009889 | regulation of biosynthetic process | 437 | 33.7 | 1.2x10^-55^ |
| GO:0010556 | regulation of macromolecule biosynthetic process | 425 | 32.8 | 4.3x10^-55^ |
| GO:0010628 | positive regulation of gene expression | 166 | 12.8 | 9.3x10^-55^ |
| GO:0051252 | regulation of RNA metabolic process | 313 | 24.2 | 2.3x10^-53^ |
| GO:0007420 | brain development | 120 | 9.3 | 5.0x10^-49^ |
| GO:0006350 | transcription | 319 | 24.6 | 6.1x10^-39^ |
| GO:0009058 | biosynthetic process | 460 | 35.5 | 1.2x10^-37^ |
| GO:0008283 | cell proliferation | 87 | 6.7 | 5.8x10^-28^ |
| GO:0030323 | respiratory tube development | 57 | 4.4 | 1.2x10^-26^ |
| GO:0030324 | lung development | 56 | 4.3 | 4.0x10^-26^ |
| GO:0007369 | gastrulation | 46 | 3.6 | 4.4x10^-26^ |
| GO:0051301 | cell division | 55 | 4.2 | 4.6x10^-5^ |
