# Supplementary material for: Properties of genes essential for mouse development
Source: PLoS One. 2017 May 31;12(5):e0178273. doi: 10.1371/journal.pone.0178273 (PMC5451031; doi:10.1371/journal.pone.0178273)
Supplement: S7 Data — (DOCX) [file pone.0178273.s007.docx]

**S7 Data.** **Top 50 enriched GO terms for viable mouse genes that are related to biological processes.**

| **GO Term ID** | **GO Term Annotation** | **Count** | **%** | **Bonferroni Corrected p-Value** |
| --- | --- | --- | --- | --- |
| GO:0050896 | response to stimulus | 977 | 28.6 | 3.2x10^-114^ |
| GO:0048518 | positive regulation of biological process | 632 | 18.5 | 1.5x10^-78^ |
| GO:0065008 | regulation of biological quality | 505 | 14.8 | 2.6x10^-71^ |
| GO:0051239 | regulation of multicellular organismal process | 388 | 11.4 | 2.7x10^-68^ |
| GO:0002376 | immune system process | 388 | 11.4 | 1.0x10^-65^ |
| GO:0048522 | positive regulation of cellular process | 546 | 16.0 | 5.0x10^-62^ |
| GO:0009605 | response to external stimulus | 326 | 9.5 | 3.7x10^-59^ |
| GO:0006950 | response to stress | 492 | 14.4 | 9.4x10^-56^ |
| GO:0048731 | system development | 722 | 21.1 | 7.7x10^-53^ |
| GO:0006955 | immune response | 253 | 7.4 | 2.0x10^-50^ |
| GO:0048583 | regulation of response to stimulus | 200 | 5.9 | 4.0x10^-50^ |
| GO:0048856 | anatomical structure development | 752 | 22.0 | 5.8x10^-50^ |
| GO:0002682 | regulation of immune system process | 195 | 5.7 | 1.1x10^-48^ |
| GO:0065007 | biological regulation | 1844 | 53.9 | 5.7x10^-45^ |
| GO:0032502 | developmental process | 877 | 25.7 | 9.5x10^-43^ |
| GO:0010941 | regulation of cell death | 271 | 7.9 | 3.1x10^-42^ |
| GO:0043067 | regulation of programmed cell death | 270 | 7.9 | 3.2x10^-42^ |
| GO:0048878 | chemical homeostasis | 202 | 5.9 | 4.8x10^-42^ |
| GO:0007610 | behavior | 216 | 6.3 | 9.3x10^-42^ |
| GO:0042592 | homeostatic process | 277 | 8.1 | 1.0x10^-41^ |
| GO:0042981 | regulation of apoptosis | 265 | 7.8 | 9.6x10^-41^ |
| GO:0007275 | multicellular organismal development | 813 | 23.8 | 2.4x10^-40^ |
| GO:0048519 | negative regulation of biological process | 496 | 14.5 | 3.1x10^-40^ |
| GO:0001775 | cell activation | 153 | 4.5 | 2.0x10^-39^ |
| GO:0042221 | response to chemical stimulus | 367 | 10.7 | 2.1x10^-39^ |
| GO:0009611 | response to wounding | 191 | 5.6 | 4.3x10^-39^ |
| GO:0032879 | regulation of localization | 209 | 6.1 | 3.5x10^-37^ |
| GO:0050865 | regulation of cell activation | 112 | 3.3 | 8.7x10^-37^ |
| GO:0001817 | regulation of cytokine production | 104 | 3.0 | 1.4x10^-36^ |
| GO:0045321 | leukocyte activation | 138 | 4.0 | 4.0x10^-36^ |
| GO:0002694 | regulation of leukocyte activation | 110 | 3.2 | 1.0x10^-35^ |
| GO:0002684 | positive regulation of immune system process | 132 | 3.9 | 1.5x10^-35^ |
| GO:0051049 | regulation of transport | 163 | 4.8 | 2.2x10^-35^ |
| GO:0048513 | organ development | 571 | 16.7 | 2.6x10^-34^ |
| GO:0010646 | regulation of cell communication | 320 | 9.4 | 4.7x10^-34^ |
| GO:0051249 | regulation of lymphocyte activation | 102 | 3.0 | 2.3x10^-32^ |
| GO:0006952 | defense response | 214 | 6.3 | 7.0x10^-32^ |
| GO:0050801 | ion homeostasis | 160 | 4.7 | 1.3x10^-31^ |
| GO:0006954 | inflammatory response | 133 | 3.9 | 1.7x10^-30^ |
| GO:0080134 | regulation of response to stress | 117 | 3.4 | 2.3x10^-29^ |
| GO:0050776 | regulation of immune response | 118 | 3.5 | 3.2x10^-29^ |
| GO:0065009 | regulation of molecular function | 239 | 7.0 | 6.8x10^-29^ |
| GO:0048523 | negative regulation of cellular process | 426 | 12.5 | 2.0x10^-28^ |
| GO:0050863 | regulation of T cell activation | 81 | 2.4 | 7.8x10^-28^ |
| GO:0046649 | lymphocyte activation | 116 | 3.4 | 1.1x10^-27^ |
| GO:0044057 | regulation of system process | 119 | 3.5 | 4.9x10^-27^ |
| GO:0048869 | cellular developmental process | 540 | 15.8 | 6.0x10^-27^ |
| GO:0019226 | transmission of nerve impulse | 128 | 3.7 | 1.1x10^-26^ |
| GO:0030154 | cell differentiation | 521 | 15.2 | 1.6x10^-26^ |
| GO:0042127 | regulation of cell proliferation | 232 | 6.8 | 1.7x10^-26^ |
