# Supplementary material for: Properties of genes essential for mouse development
Source: PLoS One. 2017 May 31;12(5):e0178273. doi: 10.1371/journal.pone.0178273 (PMC5451031; doi:10.1371/journal.pone.0178273)
Supplement: S8 Data — (DOCX) [file pone.0178273.s008.docx]

**S8 Data.** **Enriched GO terms for essential mouse genes that are related to molecular function.**

| **GO Term ID** | **GO Term Annotation** | **Count** | **%** | **Bonferroni Corrected p-Value** |
| --- | --- | --- | --- | --- |
| GO:0030528 | transcription regulator activity | 292 | 22.5 | 3.7x10^-73^ |
| GO:0003677 | DNA binding | 364 | 28.1 | 3.4x10^-72^ |
| GO:0005515 | protein binding | 740 | 57.1 | 2.5x10^-63^ |
| GO:0003700 | transcription factor activity | 212 | 16.4 | 1.8x10^-60^ |
| GO:0005488 | binding | 1107 | 85.5 | 1.3x10^-56^ |
| GO:0043565 | sequence-specific DNA binding | 161 | 12.4 | 3.1x10^-48^ |
| GO:0003676 | nucleic acid binding | 409 | 31.6 | 3.2x10^-34^ |
| GO:0016563 | transcription activator activity | 80 | 6.2 | 2.6x10^-24^ |
| GO:0003682 | chromatin binding | 58 | 4.5 | 1.4x10^-21^ |
| GO:0008134 | transcription factor binding | 75 | 5.8 | 1.0x10^-17^ |
| GO:0016564 | transcription repressor activity | 54 | 4.2 | 1.2x10^-11^ |
| GO:0003712 | transcription cofactor activity | 49 | 3.8 | 1.8x10^-11^ |
| GO:0046983 | protein dimerization activity | 67 | 5.2 | 3.5x10^-9^ |
| GO:0016773 | phosphotransferase activity, alcohol group as acceptor | 103 | 8.0 | 3.4x10^-7^ |
| GO:0004672 | protein kinase activity | 89 | 6.9 | 1.3x10^-6^ |
| GO:0032559 | adenyl ribonucleotide binding | 177 | 13.7 | 1.5x10^-6^ |
| GO:0030554 | adenyl nucleotide binding | 183 | 14.1 | 2.9x10^-6^ |
| GO:0005524 | ATP binding | 174 | 13.4 | 3.2x10^-6^ |
| GO:0001883 | purine nucleoside binding | 183 | 14.1 | 5.4x10^-6^ |
| GO:0001882 | nucleoside binding | 183 | 14.1 | 8.9x10^-6^ |
| GO:0043566 | structure-specific DNA binding | 24 | 1.9 | 9.1x10^-6^ |
| GO:0046982 | protein heterodimerization activity | 31 | 2.4 | 9.9x10^-6^ |
| GO:0016740 | transferase activity | 198 | 15.3 | 1.1x10^-5^ |
| GO:0016301 | kinase activity | 111 | 8.6 | 1.1x10^-5^ |
| GO:0003705 | RNA polymerase II transcription factor activity, enhancer binding | 15 | 1.2 | 1.4x10^-5^ |
| GO:0016772 | transferase activity, transferring phosphorus-containing groups | 123 | 9.5 | 3.3x10^-5^ |
| GO:0003713 | transcription coactivator activity | 27 | 2.1 | 3.4x10^-5^ |
| GO:0004674 | protein serine/threonine kinase activity | 65 | 5.0 | 1.9x10^-4^ |
| GO:0003702 | RNA polymerase II transcription factor activity | 26 | 2.0 | 2.1x10^-4^ |
| GO:0008013 | beta-catenin binding | 11 | 0.8 | 8.0x10^-4^ |
| GO:0010843 | promoter binding | 11 | 0.8 | 8.0x10^-4^ |
| GO:0042054 | histone methyltransferase activity | 14 | 1.1 | 8.1x10^-4^ |
| GO:0008276 | protein methyltransferase activity | 16 | 1.2 | 9.0x10^-4^ |
| GO:0004713 | protein tyrosine kinase activity | 33 | 2.5 | 0.001 |
| GO:0032553 | ribonucleotide binding | 194 | 15.0 | 0.001 |
| GO:0032555 | purine ribonucleotide binding | 194 | 15.0 | 0.001 |
| GO:0003714 | transcription corepressor activity | 20 | 1.5 | 0.002 |
| GO:0017076 | purine nucleotide binding | 200 | 15.4 | 0.002 |
| GO:0019899 | enzyme binding | 40 | 3.1 | 0.003 |
| GO:0000166 | nucleotide binding | 226 | 17.5 | 0.004 |
| GO:0008083 | growth factor activity | 28 | 2.2 | 0.012 |
| GO:0004714 | transmembrane receptor protein tyrosine kinase activity | 16 | 1.2 | 0.019 |
| GO:0008170 | N-methyltransferase activity | 14 | 1.1 | 0.021 |
| GO:0043169 | cation binding | 365 | 28.2 | 0.023 |
| GO:0046872 | metal ion binding | 362 | 28.0 | 0.023 |
| GO:0005024 | transforming growth factor beta receptor activity | 8 | 0.6 | 0.039 |
| GO:0004675 | transmembrane receptor protein serine/threonine kinase activity | 8 | 0.6 | 0.039 |
| GO:0043167 | ion binding | 367 | 28.3 | 0.041 |
| GO:0005102 | receptor binding | 83 | 6.4 | 0.047 |
| GO:0003690 | double-stranded DNA binding | 12 | 0.9 | 0.049 |
