# Supplementary material for: Properties of genes essential for mouse development
Source: PLoS One. 2017 May 31;12(5):e0178273. doi: 10.1371/journal.pone.0178273 (PMC5451031; doi:10.1371/journal.pone.0178273)
Supplement: S9 Data — (DOCX) [file pone.0178273.s009.docx]

**S9 Data. Top 50 enriched GO terms for viable mouse genes that are related to molecular function.**

| **GO Term ID** | **GO Term Annotation** | **Count** | **%** | **Bonferroni Corrected p-Value** |
| --- | --- | --- | --- | --- |
| GO:0005515 | protein binding | 1723 | 50.4 | 1.2x10^-95^ |
| GO:0005488 | binding | 2671 | 78.1 | 2.3x10^-48^ |
| GO:0005102 | receptor binding | 273 | 8.0 | 1.4x10^-29^ |
| GO:0008528 | peptide receptor activity, G-protein coupled | 76 | 2.2 | 5.8x10^-24^ |
| GO:0001653 | peptide receptor activity | 76 | 2.2 | 5.8x10^-24^ |
| GO:0042277 | peptide binding | 96 | 2.8 | 7.6x10^-24^ |
| GO:0019955 | cytokine binding | 65 | 1.9 | 1.1x10^-22^ |
| GO:0022838 | substrate specific channel activity | 162 | 4.7 | 1.6x10^-22^ |
| GO:0022803 | passive transmembrane transporter activity | 163 | 4.8 | 3.0x10^-22^ |
| GO:0015267 | channel activity | 163 | 4.8 | 3.0x10^-22^ |
| GO:0005216 | ion channel activity | 154 | 4.5 | 3.1x10^-20^ |
| GO:0022857 | transmembrane transporter activity | 281 | 8.2 | 6.1x10^-18^ |
| GO:0022836 | gated channel activity | 127 | 3.7 | 2.5x10^-17^ |
| GO:0005215 | transporter activity | 337 | 9.9 | 1.9x10^-16^ |
| GO:0022891 | substrate-specific transmembrane transporter activity | 251 | 7.3 | 1.5x10^-15^ |
| GO:0022892 | substrate-specific transporter activity | 277 | 8.1 | 5.4x10^-15^ |
| GO:0015075 | ion transmembrane transporter activity | 227 | 6.6 | 1.7x10^-14^ |
| GO:0005509 | calcium ion binding | 273 | 8.0 | 7.9x10^-14^ |
| GO:0004896 | cytokine receptor activity | 41 | 1.2 | 1.9x10^-13^ |
| GO:0022834 | ligand-gated channel activity | 61 | 1.8 | 7.6x10^-13^ |
| GO:0015276 | ligand-gated ion channel activity | 61 | 1.8 | 7.6x10^-13^ |
| GO:0004672 | protein kinase activity | 202 | 5.9 | 1.9x10^-12^ |
| GO:0005261 | cation channel activity | 107 | 3.1 | 2.3x10^-12^ |
| GO:0030594 | neurotransmitter receptor activity | 52 | 1.5 | 1.4x10^-11^ |
| GO:0042165 | neurotransmitter binding | 52 | 1.5 | 1.4x10^-11^ |
| GO:0016301 | kinase activity | 259 | 7.6 | 5.7x10^-11^ |
| GO:0005230 | extracellular ligand-gated ion channel activity | 43 | 1.3 | 5.9x10^-11^ |
| GO:0016773 | phosphotransferase activity, alcohol group as acceptor | 226 | 6.6 | 1.3x10^-10^ |
| GO:0046873 | metal ion transmembrane transporter activity | 115 | 3.4 | 2.3x10^-10^ |
| GO:0030246 | carbohydrate binding | 121 | 3.5 | 1.1x10^-9^ |
| GO:0008066 | glutamate receptor activity | 29 | 0.8 | 4.8x10^-9^ |
| GO:0005125 | cytokine activity | 79 | 2.3 | 5.6x10^-9^ |
| GO:0005262 | calcium channel activity | 41 | 1.2 | 9.5x10^-9^ |
| GO:0008289 | lipid binding | 125 | 3.7 | 3.0x10^-8^ |
| GO:0008083 | growth factor activity | 65 | 1.9 | 4.3x10^-8^ |
| GO:0046983 | protein dimerization activity | 123 | 3.6 | 4.9x10^-8^ |
| GO:0008324 | cation transmembrane transporter activity | 160 | 4.7 | 2.6x10^-7^ |
| GO:0004871 | signal transducer activity | 712 | 20.8 | 3.1x10^-7^ |
| GO:0060089 | molecular transducer activity | 712 | 20.8 | 3.1x10^-7^ |
| GO:0032403 | protein complex binding | 42 | 1.2 | 6.5x10^-7^ |
| GO:0004950 | chemokine receptor activity | 19 | 0.6 | 2.2x10^-6^ |
| GO:0008227 | amine receptor activity | 32 | 0.9 | 2.8x10^-6^ |
| GO:0005179 | hormone activity | 52 | 1.5 | 5.6x10^-6^ |
| GO:0016772 | transferase activity, transferring phosphorus-containing groups | 271 | 7.9 | 5.7x10^-6^ |
| GO:0019956 | chemokine binding | 19 | 0.6 | 8.1x10^-6^ |
| GO:0030247 | polysaccharide binding | 56 | 1.6 | 1.2x10^-5^ |
| GO:0001871 | pattern binding | 56 | 1.6 | 1.2x10^-5^ |
| GO:0003700 | transcription factor activity | 225 | 6.6 | 1.8x10^-5^ |
| GO:0008509 | anion transmembrane transporter activity | 56 | 1.6 | 2.4x10^-5^ |
| GO:0005539 | glycosaminoglycan binding | 51 | 1.5 | 2.6x10^-5^ |
